# Lipidomic profile of human nasal mucosa and associations with circulating fatty acids and olfactory deficiency

**DOI:** 10.1038/s41598-021-93817-1

**Published:** 2021-08-18

**Authors:** Spiro Khoury, Volker Gudziol, Stéphane Grégoire, Stéphanie Cabaret, Susanne Menzel, Lucy Martine, Esther Mézière, Vanessa Soubeyre, Thierry Thomas-Danguin, Xavier Grosmaitre, Lionel Bretillon, Olivier Berdeaux, Niyazi Acar, Thomas Hummel, Anne Marie Le Bon

**Affiliations:** 1 Centre des Sciences du Goût et de l’Alimentation, AgroSup Dijon, CNRS, INRAE, Université Bourgogne Franche-Comté, 21000 Dijon, France; 2 Department of Otorhinolaryngology, Interdisciplinary Center Smell and Taste, TU Dresden, Dresden, Germany

**Keywords:** Chemical biology, Neuroscience, Biomarkers

## Abstract

The nasal mucosa (NM) contains olfactory mucosa which contributes to the detection of odorant molecules and the transmission of olfactory information to the brain. To date, the lipid composition of the human NM has not been adequately characterized. Using gas chromatography, liquid chromatography coupled to mass spectrometry and thin layer chromatography, we analyzed the fatty acids and the phospholipid and ceramide molecular species in adult human nasal and blood biopsies. Saturated and polyunsaturated fatty acids (PUFAs) accounted for 45% and 29% of the nasal total fatty acids, respectively. Fatty acids of the n-6 family were predominant in the PUFA subgroup. Linoleic acid and arachidonic acid (AA) were incorporated in the main nasal phospholipid classes. Correlation analysis revealed that the nasal AA level might be positively associated with olfactory deficiency. In addition, a strong positive association between the AA levels in the NM and in plasma cholesteryl esters suggested that this blood fraction might be used as an indicator of the nasal AA level. The most abundant species of ceramides and their glycosylated derivatives detected in NM contained palmitic acid and long-chain fatty acids. Overall, this study provides new insight into lipid species that potentially contribute to the maintenance of NM homeostasis and demonstrates that circulating biomarkers might be used to predict nasal fatty acid content.

## Introduction

Olfaction, the sense of smell, is a complex sensory processing system capable of detecting and discriminating thousands of volatile chemical compounds (odorants). Olfactory signals are crucial for a wide range of activities, such as selection of palatable and nutritious foods, reproduction, sensing of predators or poisonous environments, and social interactions. Most mammals have evolved several sensory subsystems to detect olfactory stimuli, including the main olfactory system^1,2^. This system is composed of a tissue localized in the nasal cavity, the olfactory mucosa, which plays a critical role in the detection of odorants and has neural connections to a region of the forebrain, the olfactory bulb. Olfactory information is subsequently relayed to the higher cortex for cognitive processing.

The olfactory mucosa is composed of the olfactory epithelium and the underlying lamina propria. The olfactory epithelium contains several distinct cell types, including olfactory sensory neurons (OSNs) and Bowman’s gland cells that secrete mucus that covers the epithelial surface^3^. OSNs are ciliated bipolar neurons that receive external chemical signals and convert them into electrical activity. The canonical pathway of signal transduction in mammalian OSNs consists of variable components, the olfactory receptors (ORs), and constant components, such as G_olf_ heterotrimeric G-proteins, adenylyl cyclase 3, and cyclic nucleotide-gated cation channels^4,5^. These key components are localized in the plasma membrane of OSN cilia that float in the mucus.

The plasma membrane is a critical hub for signaling proteins, such as receptors that interact with external signals and amphitropic proteins that enable signal propagation. The proximity between receptors and peripheral proteins is often necessary for cellular signal transduction^6,7^. Membrane lipids facilitate these interactions and are therefore essential for many cellular activities. Breakdown products of membrane lipids also serve as second messengers involved in various cellular processes, such as inflammation, proliferation, or cell death^6,7^. Hence, better understanding of the membrane lipid composition is essential since it can influence the activity of the signaling complexes and other biological pathways.

Comprehensive lipid analyses have been recently carried out in olfactory mucosa from rodents and pigs^8,9^. These studies revealed that phospholipids (PLs) are predominant in these tissues and are characterized by species enriched in n-3 polyunsaturated fatty acids (PUFAs), including docosahexaenoic acid (DHA; 22:6n-3). Complex sphingolipids, such as gangliosides, were also identified in olfactory mucosa. The data on human olfactory mucosa are very scarce. To the best of our knowledge, only a single study examined the lipid profile of sinus mucosa from patients with chronic rhinosinusitis^10^. Cholesterol, ceramides (Cer), and some PL classes (phosphatidylcholine (PC), phosphatidylethanolamine (PE), and sphingomyelin (SM)) were detected in sinus mucosa^10^.

The objectives of the present study were to expand our knowledge about the lipid composition of human nasal mucosa (NM) and to investigate whether nasal fatty acid (FA) levels can be predicted by blood FA amounts. To achieve the first aim, we performed an in-depth analysis of the FAs and of the PL and Cer molecular species of nasal biopsies using gas chromatography (GC) and liquid chromatography coupled to mass spectrometry (MS), respectively. In adult humans, there is no clear-cut border between olfactory and respiratory mucosa. As a matter of fact, the olfactory mucosa is frequently disrupted with interspersed patches of respiratory mucosa^11–13^. Therefore, it is worth mentioning that our analyses were carried out on nasal biopsies that may contain both types of mucosa. To achieve the second aim, we analyzed the FA profiles of blood fractions (total plasma, plasma PL, plasma cholesteryl esters (EC) and erythrocytes) from human donors and we assessed possible relationships between circulating and nasal FA levels. In addition, we examined potential associations between nasal FA levels and individual characteristics of the subjects (including smoking status, body mass index (BMI), and olfactory abilities).

## Results

### Clinical characteristics of the patients

Clinical characteristics of human subjects are presented in Table 1. The median age of the donors was 46 years (interquartile range = 34–52.5). Seven patients were smokers, and 4 patients had a BMI > 30. Assessment of olfactory performance by the ‘‘Sniffin’ Sticks’’ test revealed that 7 patients were hyposmic. The total cholesterol concentration in the plasma ranged from 0.72 to 2.22 mg/mL (median concentration = 1.51 mg/mL).

**Table 1:** Individual characteristics of human donors.

Patient	Sex	Age	BMI	Smoking status	Olfactory diagnosis	Plasma total cholesterol (mg/mL)
1	Male	29	24.8	Non-smoker	Normosmia	1.80
2	Male	38	23.4	Non-smoker	Hyposmia	1.58
3	Female	53	25.6	Non-smoker	Normosmia	1.58
4	Female	36	24.3	Smoker	Normosmia	1.31
5	Female	61	29.4	Non-smoker	Normosmia	1.54
6	Male	47	35.2	Non-smoker	Normosmia	1.31
7	Male	29	19.9	Smoker	Hyposmia	1.34
8	Male	49	35.0	Non-smoker	Normosmia	1.48
9	Female	49	25.5	Smoker	Normosmia	1.89
10	Male	21	19.0	Smoker	Normosmia	0.86
11	Male	18	23.5	Smoker	Normosmia	0.72
12	Male	52	24.7	Non-smoker	Hyposmia	1.39
13	Female	76	24.3	Non-smoker	Normosmia	1.76
14	Male	33	32.3	Non-smoker	Hyposmia	1.65
15	Female	80	26.8	Non-smoker	Normosmia	1.35
16	Male	46	26.1	Non-smoker	Normosmia	2.22
17	Male	20	29.4	Non-smoker	Normosmia	1.51
18	Female	49	29.1	Non-smoker	Hyposmia	1.96
19	Female	36	23.9	Non-smoker	Normosmia	1.41
20	Male	35	27.8	Smoker	Hyposmia	1.83
21	Female	39	19.7	Non-smoker	Hyposmia	1.28
22	Male	62	33.1	Smoker	Normosmia	1.55
23	Male	80	23.9	Non-smoker	Normosmia	1.36
Median		46	25.5			1.51
IQR		34–52.5	23.9–29.2			1.34–1.70
Range		18–80	19–35.2			0.72–2.22

*BMI* body mass index, *IQR* interquartile range (Q1–Q3).

### Lipid composition of nasal mucosa

#### Fatty acid composition

**Figure 1:**
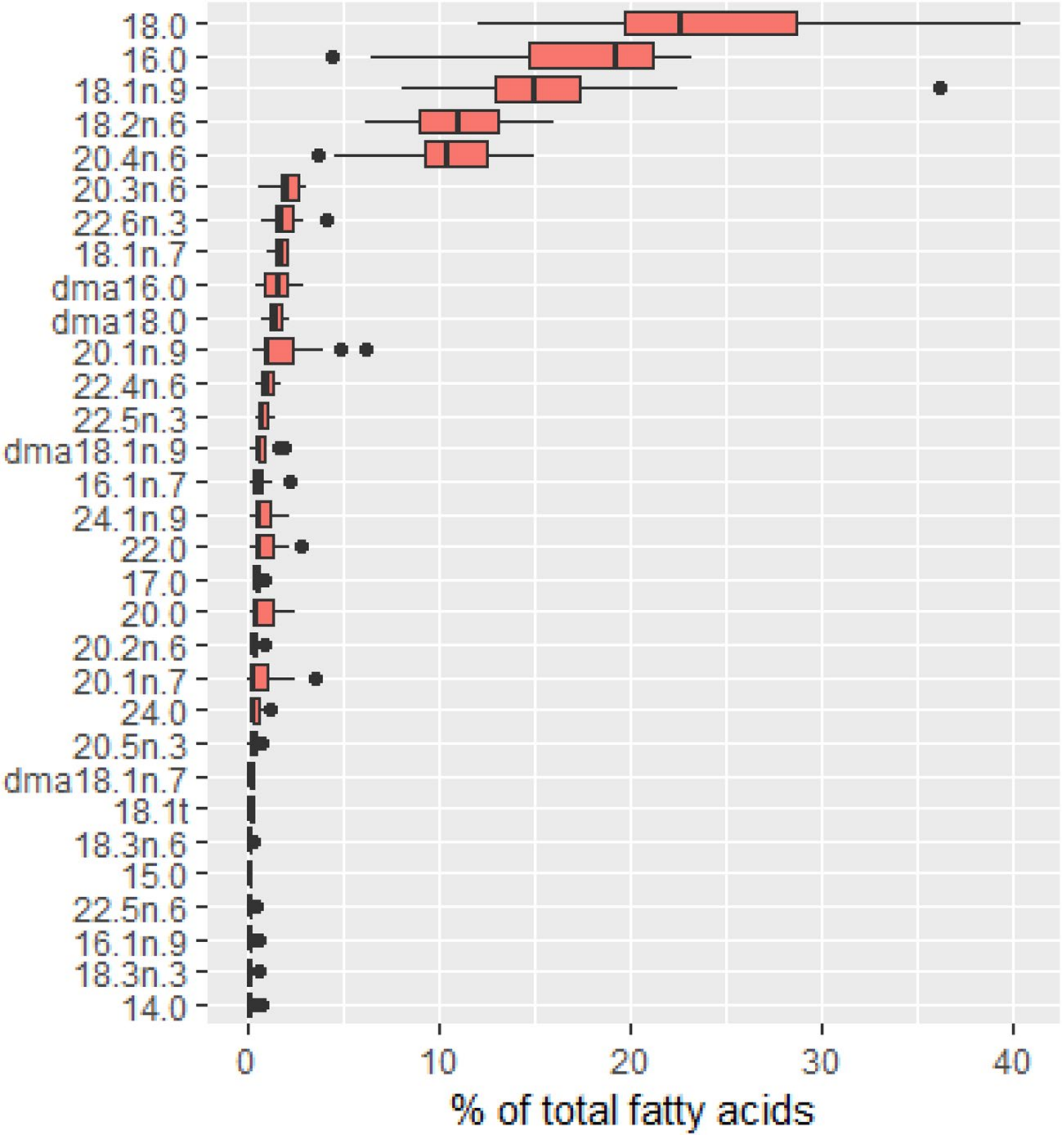
Box plot representation of fatty acid levels in human nasal mucosa**.** The results are presented as the median, first and third quartiles, and range (n = 20).

Saturated FAs (SFAs) represented approximately 45% of all FAs and were the main FAs detected in nasal biopsies (Table 2). Palmitic (16:0) and stearic (18:0) acids were the main SFAs. Nearly equivalent levels of monounsaturated FAs (MUFAs) and PUFAs were detected in human NM (~ 22% and ~ 29% of total FAs, respectively). Oleic acid (18:1n-9) was the main MUFA identified in this tissue (~ 16% of total FAs). n-6 PUFAs accounted for ca. 88% of total PUFAs. Linoleic acid (LA; 18:2n-6) and arachidonic acid (AA; 20:4n-6) were the most important n-6 PUFAs. n-3 PUFAs represented an average of 3.4% of total FAs. DHA (22:6n-3) and, to a lesser extent, docosapentaenoic acid (22:5n-3) were the principal compounds in the n-3 PUFA group. Dimethyl acetals (DMAs), that are alkenyl moieties derived only from plasmalogens, represented an average of 4.2% of all FAs. The amount of DMAs is known to reflect half of the plasmalogen pool in PL-rich tissues^14^; thus, plasmalogens accounted for approximately 8.4% of PLs in human NM. The levels of the most abundant FAs (especially 16:0, 18:0, and 18:1n-9) were highly variable (Fig. 1).

**Table 2:** Fatty acid composition of total lipids from human nasal mucosa (% of total fatty acids).

Fatty acids	Mean ± sem^a^	Median	[IQR]
14:0	0.17 ± 0.04	0.13	[0.09–0.18]
15:0	0.21 ± 0.02	0.19	[0.13–0.24]
dma16:0	1.58 ± 0.17	1.68	[0.94–2.05]
16:0	17.30 ± 1.20	19.31	[14.73–21.16]
16:1n-9	0.22 ± 0.03	0.16	[0.15–0.22]
16:1n-7	0.69 ± 0.11	0.65	[0.38–0.84]
17:0	0.56 ± 0.03	0.56	[0.50–0.60]
dma18:0	1.56 ± 0.10	1.56	[1.29–1.84]
dma18:1n-9	0.81 ± 0.11	0.69	[0.57–0.86]
dma18:1n-7	0.25 ± 0.02	0.26	[0.21–0.30]
18:0	24.20 ± 1.62	22.64	[19.73–28.73]
18:1t	0.22 ± 0.02	0.21	[0.17 -0.28]
18:1n-9	15.71 ± 1.33	15.07	[12.97 -17.31]
18:1n-7	1.79 ± 0.08	1.85	[1.46–2.12]
18:2n-6 (LA)	11.04 ± 0.69	11.11	[8.95–13.07]
20:0	0.93 ± 0.17	0.52	[0.39–1.30]
18:3n-6	0.20 ± 0.02	0.20	[0.13–0.22]
20:1n-9	1.92 ± 0.37	1.09	[0.86–2.39]
18:3n-3 (ALA)	0.17 ± 0.03	0.15	[0.10–0.18]
20:1n-7	0.85 ± 0.21	0.39	[0.20–1.14]
20:2n-6	0.43 ± 0.03	0.41	[0.34–0.50]
22:0	0.99 ± 0.17	0.59	[0.50–1.39]
20:3n-6	2.16 ± 0.14	2.08	[1.83–2.66]
20:4n-6 (AA)	10.19 ± 0.68	10.48	[9.26–12.58]
20:5n-3	0.36 ± 0.04	0.32	[0.24–0.44]
24:0	0.51 ± 0.08	0.36	[0.28–0.63]
24:1n-9	0.91 ± 0.14	0.61	[0.49–1.26]
22:4n-6	1.04 ± 0.08	1.02	[0.74–1.30]
22:5n-6	0.19 ± 0.02	0.18	[0.14–0.22]
22:5n-3 (n-3 DPA)	0.86 ± 0.07	0.84	[0.65–1.04]
22:6n-3 (DHA)	1.99 ± 0.18	1.88	[1.48–2.38]
Total SFAs	44.86 ± 1.19	43.67	[42.74–45.57]
Total MUFAs	22.31 ± 1.30	20.72	[19.57–22.68]
Total PUFAs	28.63 ± 1.32	30.26	[25.91–32.23]
Total dmas	4.20 ± 0.23	4.35	[3.63–4.95]
Total n-3	3.38 ± 0.23	3.04	[2.59–3.99]
Total n-6	25.26 ± 1.28	26.56	[23.22–28.62]
n-6/n-3	8.02 ± 0.57	7.89	[6.66–9.76]

*IQR* interquartile range (Q1–Q3), *AA* arachidonic acid, *ALA* α-linolenic acid, *DHA* docosahexaenoic acid, *dma* dimethyl acetal, *DPA* docosapentaenoic acid, *LA* linoleic acid, *MUFAs* monounsaturated fatty acids, *PUFAs* polyunsaturated fatty acids, *SFAs* saturated fatty acids.

^a^n = 20 subjects.

#### Phospholipid classes

**Figure 2:**
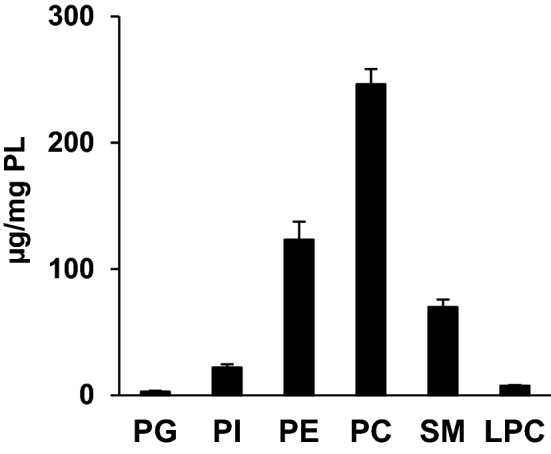
Phospholipid content of human nasal mucosa. Values are presented as the mean ± sem (n = 19). *PG* phosphatidylglycerol, *PI* phosphatidylinositol, *PE* phosphatidylethanolamine, *PC* phosphatidylcholine, *SM* sphingomyelin, *LPC* lysophosphatidylcholine.

High performance liquid chromatography (HPLC) under the hydrophilic interaction liquid chromatography (HILIC) conditions was used to separate and detect PL classes. PC and, to a lesser extent, PE were the most abundant PL classes detected in human NM (Fig. 2). Substantial amounts of SM and phosphatidylinositol (PI) and lower quantities of lysophosphatidylcholine (LPC) and phosphatidylglycerol (PG) were also detected. The phosphatidylserine (PS) class could not be detected in human NM by HPLC coupled to Corona-CAD. However, PS molecular species were identified and quantified by HPLC coupled to mass spectrometry (MS), as shown below.

#### Phospholipid species

**Figure 3:**
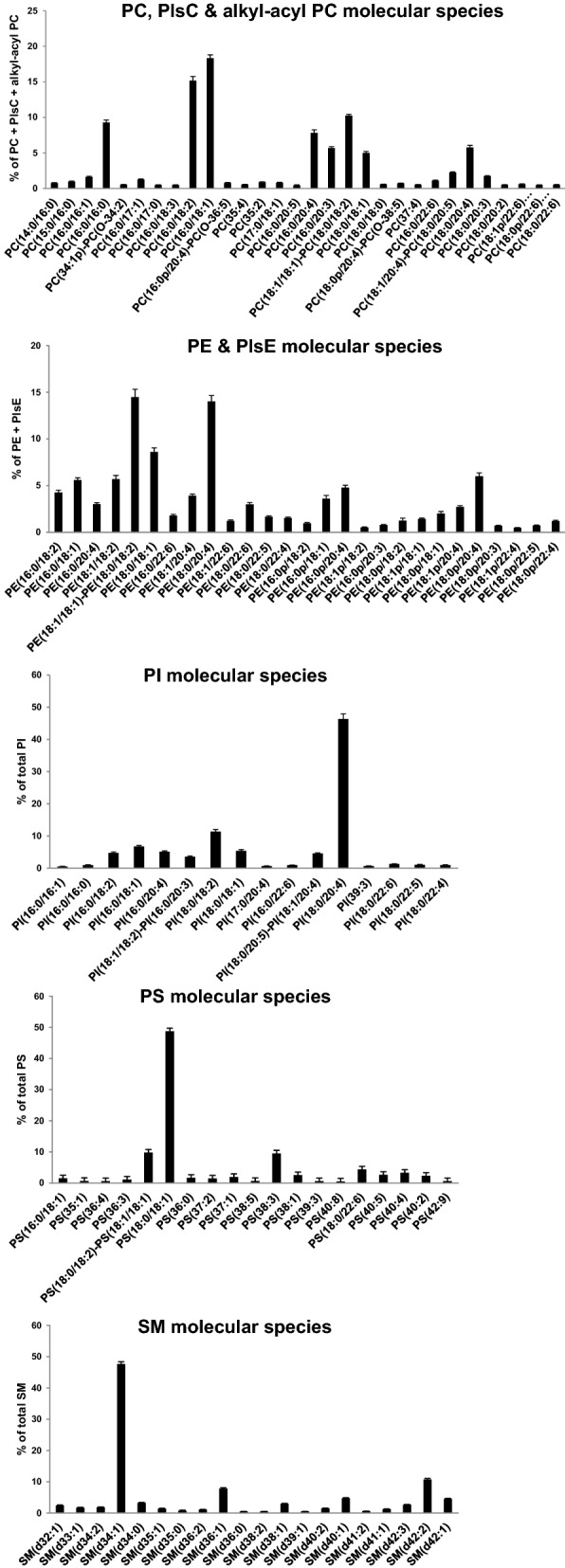
Concentrations of individual phospholipid species identified in human nasal mucosa. Values are presented as the mean ± sem (n = 23). Only species with levels > 0.5% are shown. Complete data are provided in Supplementary Table S1.

HPLC coupled to MS was used to identify and quantify molecular species of various PL classes in human NM samples. PL species composition was expressed relative to the total lipids in each PL class. The main species are shown in Fig. 3**;** all data are presented in Supplementary Table S1.

In the PC class, the most represented species were PC 16:0/18:1 and PC 16:0/18:2. Substantial amounts of PC 16:0/16:0, PC 16:0/20:4, 18:0/18:1, 36:2 (including a mixture of 18:0/18:2 and 18:1/18:1), and 18:0/20:4 were also detected. Plasmenylcholine (PlsC) and alkyl-acyl PC molecules were also detected in nasal samples and accounted for 4.24% of the PC class. Since these species were detected with very low abundances at the same exact masses, they were not distinguishable under our experimental conditions. Both PlsC and alkyl-acyl PC molecules are listed in Supplementary Table S1. The main PlsC and alkyl-acyl PC species were PC 16:0p/20:4 and/or PC O-36:5, as well as PC 18:0p/20:4 and/or PC O-38:5.

In the PE class, PE 36:2 (represented by a mixture of PE 18:1/18:1 and PE 18:0/18:2 species) and PE 18:0/20:4 were highly represented in human NM. In addition, the PE class included substantial levels of PE 18:0/18:1, PE 18:1/18:2, and PE 16:0/18:1. Various plasmenylethanolamine (PlsE) species were also detected in human NM. Targeted analysis (multiple reaction monitoring mode) using triple quadrupole mass spectrometer enabled them to be distinguished from corresponding alkyl-acyl PE and other PE species. These PlsE compounds accounted for 28% of the PE class. The main PlsE species were 16:0p/20:4, 16:0p/18:1, and 18:0p/20:4. The most abundant molecular species of the PI class was PI 18:0/20:4. A substantial level of PI 18:0/18:2 was also detected in this PL class. The PS class of human NM was characterized by a predominance of PS 18:0/18:1. Moderate levels of PS 36:2 represented by a mixture of PS 18:0/18:2 and PS 18:1/18:1 species and PS 38:3 were also detected in this class. SM d34:1 was the major entity of the SM class. This class also contained notable levels of SM d42:2 and SM d36:1. LPC 16:0 (44% of total LPCs) was the main LPC detected in human NM. LPC 18:0, LPC 18:1, LPC 18:2, and LPC 20:4 were also detected (Supplementary Table S1).

#### Ceramide and glycosphingolipid species

**Figure 4:**
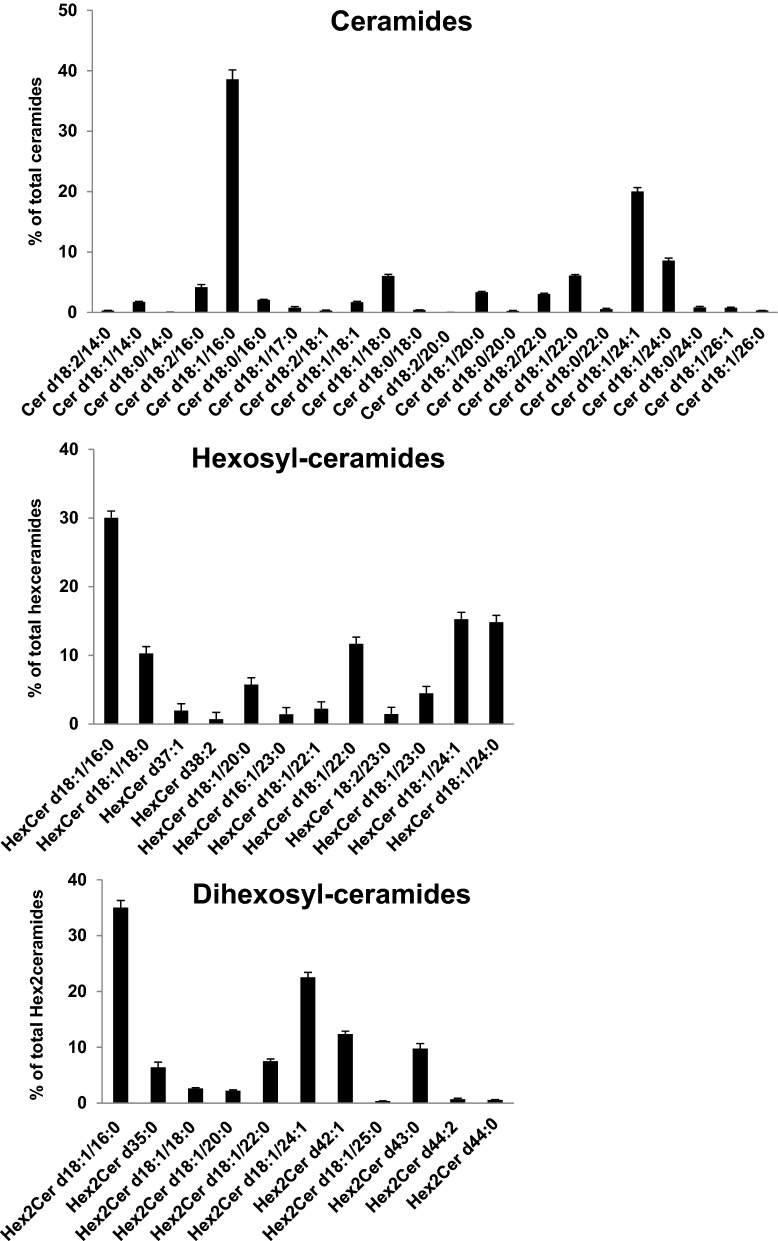
Concentrations of individual ceramide, hexosyl-ceramide and dihexosyl-ceramide species identified in human nasal mucosa. Values are presented as the mean ± sem (n = 23).

HPLC under the HILIC conditions was used to separate the Cer and glycosphingolipid (hexosyl-ceramides (HexCer), and dihexosyl-ceramides (Hex2Cer)) classes from the human NM lipid extract based on an increase in polarity. Coupling of this method to a high-resolution mass spectrometer (Orbitrap Fusion) enabled identification of various molecular species of these lipid classes. The relative distribution of these molecular species is presented in Fig. 4. The Cer class exhibited the highest diversity of molecular species and included 22 identified species. The glycosphingolipid profile was characterized by a lower diversity than that of the Cer class and included 12 and 11 molecular species of HexCer and Hex2Cer classes, respectively. Species composed of sphingosine (d18:1) and palmitic acid (16:0) were the most abundant in the Cer class and in both glycosphingolipid classes. The composition of the most abundant species of these molecules, concerning the sphingosine and the fatty acid, was similar to that of the most abundant species of SM class (d34:1) as shown in Fig. 3. Substantial levels of d18:1/24:1 and d18:1/24:0 (or d41:1) were also detected in the Cer class and in glycosphingolipids.

### Fatty acid composition of the blood samples

The complete FA compositions of plasma, plasma-PL, plasma-cholesteryl esters (plasma-CE), and erythrocytes obtained by standard GC are presented in Table 3. The plasma samples were characterized by high levels of palmitic acid (16:0), oleic acid (18:1n-9), and LA (18:2n-6) and by moderate levels of stearic acid (18:0) and AA (20:4n-6). PUFAs of the n-3 family were present at lower levels, and DHA (22:6n-3) was the most abundant. A very high level of 16:0 and lower levels of 18:0, 18:1n-9, LA, and AA were detected in PL extracted from the plasma (plasma-PL). n-3 PUFAs accounted for approximately 5% of total FAs. The CE samples extracted from the plasma (plasma-CE) were characterized by a very high level of LA and by lower levels of 18:1n-9, 16:0, and AA. Finally, erythrocytes were found to contain high levels of 16:0, 18:0, and 18:1n-9 and lower levels of LA and AA. Notably, variability of the FA profiles in the blood compartments was lower than that in the nasal tissue.

**Table 3:** Fatty acid composition of total lipids from erythrocytes, plasma, plasma phospholipids, and plasma ester cholesterol (% of total fatty acids).

Fatty acids	Plasma		Plasma-PL		Plasma-EC		Erythrocytes	
	Mean ± sem^a^	Median [IQR]	Mean ± sem	Median [IQR]	Mean ± sem	Median [IQR]	Mean ± sem	Median [IQR]
14:0	0.70 ± 0.04	0.69 [0.57–0.77]	0.31 ± 0.02	0.30 [0.27–0.35]	0.89 ± 0.07	0.92 [0.63–1.13]	0.47 ± 0.03	0.46 [0.34–0.57]
15:0	0.24 ± 0.01	0.23 [0.19–0.27]	0.23 ± 0.01	0.23 [0.19–0.26]	0.24 ± 0.01	0.24 [0.20–0.28]	0.25 ± 0.01	0.24 [0.19–0.31]
dma16:0	0.32 ± 0.02	0.32 [0.26–0.37]	0.71 ± 0.04	0.71 [0.62–0.79]			1.92 ± 0.08	1.91 [1.83–2.07]
16:0	22.42 ± 0.34	22.19 [21.76–23.01]	29.39 ± 0.39	29.58 [28.10–30.35]	13.28 ± 0.47	12.78 [12.46–13.46]	27.55 ± 0.76	27.64 [25.39–30.00]
16:1n-9	0.38 ± 0.02	0.36 [0.32–0.42]	0.16 ± 0.01	0.14 [0.12–0.18]	0.46 ± 0.02	0.45 [0.39–0.51]	0.12 ± 0.01	0.12 [0.11–0.13]
16:1n-7	2.23 ± 0.16	2.09 [1.96–2.38]	0.62 ± 0.04	0.62 [0.50–0.70]	3.00 ± 0.26	2.77 [2.23–3.44]	0.55 ± 0.06	0.52 [0.40–0.62]
17:0	0.30 ± 0.01	0.29 [0.26–0.32]	0.42 ± 0.02	0.40 [0.38–0.46]	0.13 ± 0.01	0.12 [0.11–0.15]	0.45 ± 0.02	0.43 [0.40–0.52]
dma18:0	0.23 ± 0.01	0.22 [0.18–0.2]	0.43 ± 0.02	0.41 [0.38–0.48]			3.03 ± 0.12	3.01 [2.86–3.27]
dma18:1n-9	0.25 ± 0.01	0.25 [0.24–0.27]	0.27 ± 0.01	0.27 [0.24–0.32]			0.61 ± 0.04	0.58 [0.52–0.69]
dma18:1n-7							0.15 ± 0.01	0.13 [0.10–0.16]
18:0	7.06 ± 0.30	6.79 [6.35–7.23]	15.25 ± 0.49	14.38 [14.01–15.41]	1.40 ± 0.09	1.24 [1.18–1.40]	20.55 ± 0.52	20.46 [19.14–21.58]
18:1t	0.71 ± 0.06	0.54 [0.49–0.86]	0.62 ± 0.05	0.52 [0.47–0.74]	0.14 ± 0.01	0.12 [0.10–0.18]	0.73 ± 0.05	0.70 [0.59–0.87]
18:1n-9	23.38 ± 0.63	23.13 [20.97–25.00]	11.52 ± 0.43	11.60 [10.76–12.21]	22.07 ± 0.65	22.29 [20.70–23.13]	16.78 ± 0.48	16.94 [14.85–18.72]
18:1n-7	1.74 ± 0.05	1.74 [1.62–1.84]	1.55 ± 0.05	1.55 [1.36–1.67]	1.36 ± 0.06	1.36 [1.16–1.47]	1.27 ± 0.05	1.27 [1.13–1.41]
18:2n-6 (LA)	26.93 ± 0.73	27.10 [25.99–28.63]	18.42 ± 0.45	18.46 [17.62–19.72]	48.73 ± 0.94	49.15 [47.71–51.64]	8.44 ± 0.35	7.92 [7.28–9.62]
20:0	0.13 ± 0.01	0.11 [0.11–0.14]	0.22 ± 0.01	0.22 [0.20–0.25]			0.30 ± 0.01	0.27 [0.24–0.35]
18:3n-6	0.36 ± 0.03	0.34 [0.29–0.44]	0.08 ± 0.01	0.08 [0.06–0.10]	0.75 ± 0.06	0.74 [0.54–0.88]		
20:1n-9	0.20 ± 0.01	0.19 [0.17–0.21]	0.20 ± 0.01	0.18 [0.16–0.21]			0.31 ± 0.02	0.29 [0.25–0.38]
18:3n-3 (ALA)	0.65 ± 0.05	0.65 [0.53–0.73]	0.22 ± 0.02	0.21 [0.17–0.24]	0.52 ± 0.03	0.50 [0.45–0.62	0.14 ± 0.02	0.12 [0.09–0.17]
20:2n-6	0.17 ± 0.01	0.16 [0.15–0.20]	0.31 ± 0.01	0.31 [0.28–0.32]			0.21 ± 0.01	0.21 [0.20–0.23]
20:3n-9	0.14 ± 0.01	0.13 [0.11–0.19]	0.18 ± 0.01	0.1 6[0.13–0.23]				
22:0	0.19 ± 0.02	0.18 [0.15–0.21]	0.45 ± 0.02	0.44 [0.42–0.49]			0.67 ± 0.03	0.64 [0.58–0.77]
20:3n-6	1.45 ± 0.08	1.49 [1.22–1.69]	2.97 ± 0.18	3.05 [2.56–3.35]	0.75 ± 0.03	0.75 [0.64–0.85]	0.91 ± 0.04	0.94 [0.75–1.07]
22:1n-9							0.12 ± 0.02	0.10 [0.09–0.12]
20:4n-6 (AA)	6.03 ± 0.40	5.96 [5.08–7.02]	9.13 ± 0.56	9.30 [7.60–10.84]	5.27 ± 0.38	5.46 [4.41–5.97]	6.87 ± 0.64	5.59 [4.91–7.82]
20:5n-3	0.87 ± 0.15	0.63 [0.54–0.79]	1.08 ± 0.18	0.86 [0.61–0.99]	0.65 ± 0.11	0.53 [0.33–0.71]	0.51 ± 0.09	0.41 [0.26–0.61]
24:0	0.20 ± 0.01	0.18 [0.17–0.22]	0.32 ± 0.02	0.32 [0.29–0.38]			1.68 ± 0.10	1.60 [1.26–1.97]
24:1n-9	0.36 ± 0.03	0.35 [0.29–0.42]	0.75 ± 0.03	0.71 [0.66–0.88]			1.85 ± 0.08	1.81 [1.60–2.14]
22:4n-6	0.17 ± 0.01	0.15 [0.14–0.18]	0.28 ± 0.03	0.27 [0.24–0.31]			1.17 ± 0.14	0.82 [0.71–1.60]
22:5n-6	0.16 ± 0.01	0.15 [0.12–0.17]	0.25 ± 0.02	0.24 [0.20–0.28]			0.22 ± 0.02	0.19 [0.15–0.26]
22:5n-3 (n-3 DPA)	0.46 ± 0.03	0.50 [0.40–0.55]	0.80 ± 0.05	0.82 [0.76–0.90]			0.80 ± 0.11	0.66 [0.46–0.90]
22:6n-3 (DHA)	1.57 ± 0.14	1.46 [1.26–1.89]	2.85 ± 0.22	2.74 [2.36–3.43]	0.35 ± 0.03	0.36 [0.27–0.45]	1.37 ± 0.17	1.14 [0.87–1.5]
Total SFAs	31.23 ± 0.59	30.68 [30.13–31.54]	46.61 ± 0.74	45.85 [44.77–46.59]	15.94 ± 0.60	15.23 [14.87–16.23]	51.92 ± 1.28	51.96 [48.63–55.23]
Total MUFAs	29.00 ± 0.72	29.30 [26.03–30.59]	15.42 ± 0.51	15.69 [14.34–16.06]	27.03 ± 0.80	27.05 [25.04–28.28]	21.73 ± 0.61	22.04 [19.35–23.93]
Total PUFAs	38.97 ± 1.17	38.11 [36.93–42.71]	36.56 ± 1.09	37.82 [36.74–39.05]	57.02 ± 1.23	58.01 [55.06–59.34]	20.65 ± 1.38	17.87 [15.83–23.98]
Total dmas	0.80 ± 0.03	0.79 [0.70–0.87]	1.41 ± 0.08	1.40 [1.22–1.59]			5.70 ± 0.22	5.64 [5.35–6.23]
Total n-3	3.55 ± 0.32	3.23 [2.83–3.98]	4.94 ± 0.40	4.68 [4.12–5.68]	1.52 ± 0.15	1.44 [1.06–1.79]	2.83 ± 0.33	2.43 [1.78–3.17]
Total n-6	35.27 ± 1.07	35.01 [33.37–37.92]	31.44 ± 0.92	32.21 [30.02–33.76]	55.50 ± 1.22	56.36 [53.81–57.43]	17.82 ± 1.10	15.81 [14.09–21.31]
n-6/n-3	11.84 ± 1.18	10.73 [9.12–12.40]	7.40 ± 0.63	6.83 [6.04–8.01]	43.59 ± 4.25	41.97 [32.00–49.13]	7.37 ± 0.62	6.36 [5.65–8.33]

*IQR* interquartile range (Q1–Q3), *AA* arachidonic acid, *ALA* α-linolenic acid, *DHA* docosahexaenoic acid, *dmas* dimethyl acetals, *DPA* docosapentaenoic acid, *EC* ester cholesterol, *LA* linoleic acid, *MUFAs* monounsaturated fatty acids, *PL* phospholipid, *PUFAs* polyunsaturated fatty acids, *SFAs* saturated fatty acids.

^a^n = 23 subjects.

### Associations between nasal and blood fatty acids

All Spearman correlation coefficients between nasal and blood FAs are shown in Supplementary Table S2. The main statistically significant associations are presented in Table 4. The most interesting results concerned nasal AA, which was positively associated with AA in the plasma-CE (*r(20)* = 0.68, *p* = 0.001), plasma-PL (*r(20)* = 0.49, *p* = 0.029), and plasma (*r(20)* = 0.48, *p* = 0.032) and with 22:4n-6 in the plasma (*r(20)* = 0.45, *p* = 0.048). Conversely, nasal AA was negatively associated with ALA in the plasma, plasma-PL, and plasma-CE with moderate correlation. Nasal oleic acid (18:1n-9), the main MUFA identified in NM, was moderately correlated with individual n-3 PUFAs (18:3n-3, 20:5n-3, and 22:6n-3) in the plasma, plasma-PL, and plasma-CE. ALA (18:3n-3) in NM was moderately correlated with EPA (20:5n-3) in the plasma and plasma-CE. DHA was negatively associated with 20:3n-9 in the plasma, with saturated FAs (20:0, 22:0 and 24:0) in the plasma, and with 18:0 in the plasma-CE, all with moderate correlations.

**Table 4:** Main significant associations between nasal and plasma fatty acid levels (Spearman test; n = 20).

Nasal FAs		Plasma	Plasma-PL	Plasma-CE
		rSpearman (P value)		
20:4n-6 (AA)	18:1n-9		−0.52 (0.019)	
	18:3n-3	−0.58 (0.009)	−0.56 (0.011)	−0.49 (0.028)
	20:4n-6	0.48 (0.032)	0.49 (0.029)	0.68 (0.001)
	22:4n-6	0.45 (0.048)		
	24:0		0.61 (0.005)	
18:1n-9	18:3n-3	0.65 (0.003)		0.54 (0.016)
	20:5n-3	0.61 (0.005)	0.69 (0.001)	0.47 (0.036)
	22:6n-3	0.52 (0.020)	0.60 (0.006)	
	n-6/n-3	−0.73 (4.10^–4^)	−0.71 (0.001)	−0.53 (0.017)
18:3n-3 (ALA)	20:0		−0.46 (0.044)	
	22:0	−0.46 (0.042)	−0.62 (0.005)	
	20:5n-3	0.48 (0.033)		0.63 (0.003)
	n-6/n-3	-0.46 (0.044)		−0.54 (0.016)
22:6n-3 (DHA)	18:0			−0.62 (0.004)
	20:0	−0.60 (0.006)		
	20:3n-9	−0.45 (0.048)		
	22:0	−0.60 (0.006)		
	24:0	−0.53 (0.019)		

*AA* arachidonic acid, *ALA* α-linolenic acid, *DHA* docosahexaenoic acid, *CE* cholesterol ester, *PL* phospholipid.

Additionally, we evaluated correlations between nasal AA-containing PL species and blood n-6 PUFAs and several significant moderate associations were found. Analysis showed that four PL species, including PE(16:0/20:4), PE(18:1/20:4), PC(16:0/20:4), and PI(16:0/20:4), were positively correlated with plasma AA (Table 5). Three of these species (PE(16:0/20:4), PE(18:1/20:4), and PC(16:0/20:4)) were also positively associated with AA in the plasma-CE. Additionally, a significant association between PE(18:1/20:4) and AA in the plasma-PL was observed. Two PI species, PI(18:0p/20:4) and PI(17:0/20:4), were positively correlated with LA in the plasma-PL and in plasma, respectively. In contrast, two PlsC species, PC(16:0p/20:4) and PC(18:0p/20:4), were negatively associated with LA in erythrocytes and in plasma-PL, respectively. A negative correlation was also observed between PI(18:0/20:5)-PI(18:1/20:4) and 22:4n-6 in the plasma and plasma-PL.

**Table 5:** Significant associations between nasal arachidonic acid (20:4)-containing phospholipid species and blood n-6 polyunsaturated fatty acids (Spearman test; n = 23).

Nasal PL species	Erythrocyte 18:2n-6	Plasma 18:2n-6	Plasma 20:2n-6	Plasma 20:4n-6	Plasma 22:4n-6	Plasma PL 18:2n-6	Plasma PL 20:4n-6	Plasma PL 22:4n-6	Plasma CE 20:3n-6	Plasma CE 20:4n-6
	rSpearman (P values)									
PE(16:0/20:4)				0.47 (0.030)						0.55 (0.008)
PE(18:1/20:4)				0.54 (0.011)			0.49 (0.021)			0.60 (0.004)
PC(16:0p/20:4)	−0.45 (0.037)									
PC(16:0/20:4)				0.43 (0.048)						0.56 (0.008)
PC(18:0p/20:4)			−0.45 (0.038)			−0.47 (0.027)				
PC(18:1/20:4)-PC(18:0/20:5)						−0.53 (0.013)				
PI(16:0/20:4)				0.45 (0.036)						
PI(18:0p/20:4)						0.45 (0.037)				
PI(17:0/20:4)		0.47 (0.029)							−0.44 (0.040)	
PI(18:0/20:5)-PI(18:1/20:4)					−0.47 (0.031)			−0.50 (0.018)		

*CE* cholesterol ester, *PC* phosphatidylcholine, *PE* phosphatidylethanolamine, *PI* phosphatidylinositol, *PL* phospholipid.

### Associations between nasal FAs and individual characteristics

**Figure 5:**
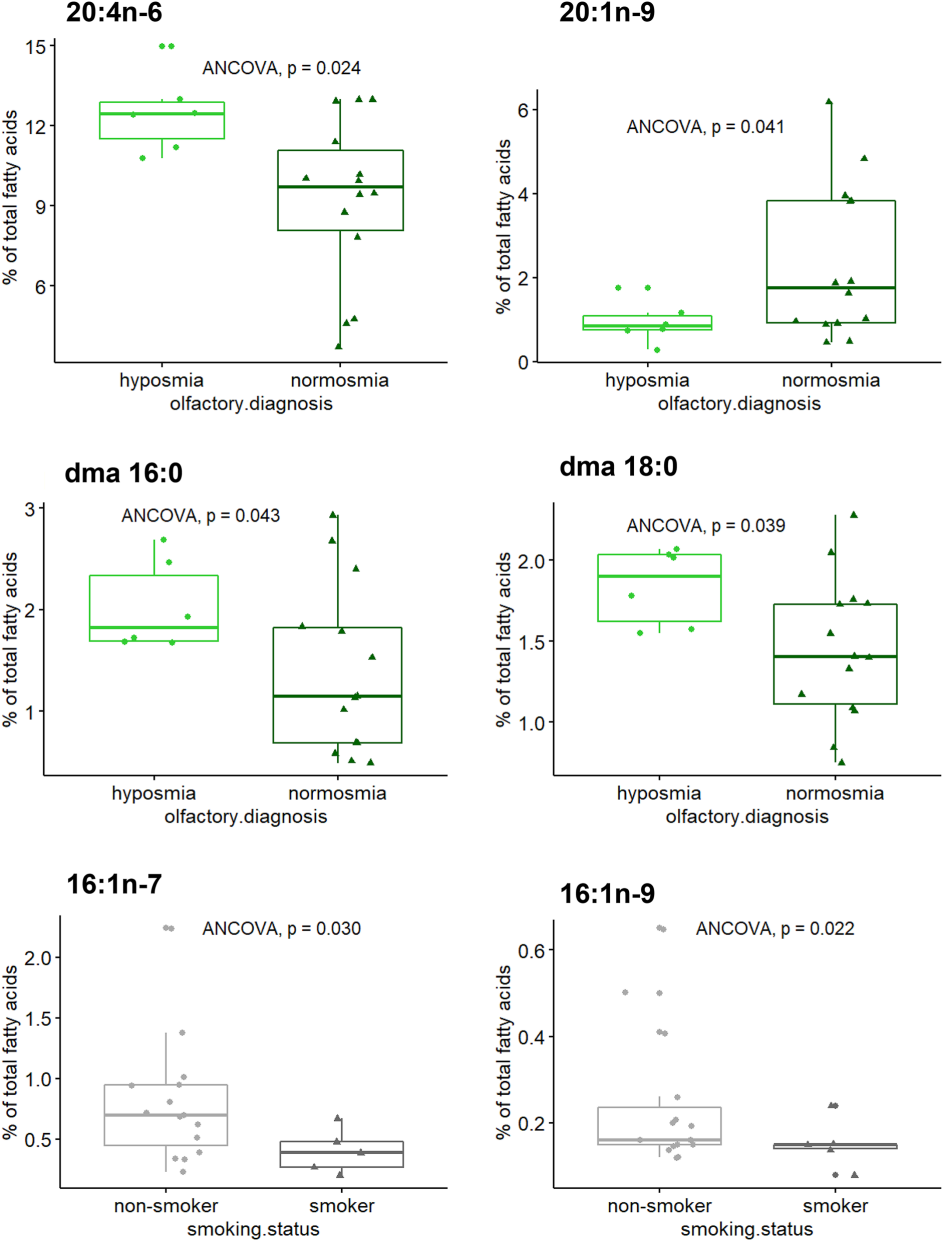
Significant associations between individual factors and nasal fatty acids estimated by type II ANCOVA. The results are presented as the median, first and third quartiles, and range (n = 20). *dma* dimethyl acetal.

Several significant associations between nasal FAs and individual characteristics were observed (Supplementary Fig. S1). After adjustment for covariates (age, sex, smoking status, BMI, and olfactory diagnosis), nasal AA, dma 16:0 and dma 18:0 levels were significantly greater in hyposmic subjects than those in normosmic subjects (Fig. 5). Conversely, the nasal 20:1n-9 level was lower in hyposmic subjects. On the other hand, smoking subjects displayed significantly reduced levels of nasal 16:1n-9 and 16:1n-7 compared to those in nonsmokers.

## Discussion

The present study aimed to describe the lipid composition of human NM. We analyzed the complete FA profile of this tissue and the composition of three major lipid groups (PL, Cer, and glycosphingolipids). In addition, we identified significant associations between nasal and blood FAs and between nasal FAs and individual characteristics of the donors.

Our data showed the presence of substantial amounts of n-6 PUFAs (mainly LA and AA) in nasal biopsies and indicated that a high level of nasal AA might be associated with hyposmia (p = 0.024). LA, the precursor of the n-6 family, and AA, a long-chain member of the n-6 family, were detected in equivalent amounts. In contrast to LA, AA is relatively rare in the diet. AA is generated from LA by consecutive elongation and desaturation catalyzed by delta-5- and delta-6-desaturases (FADS1 and FADS2) and an elongase (ELOVL5)^15^. The liver is the primary site for PUFA metabolism; however, it can also take place in other tissues^16,17^. Several desaturases and elongases have been detected in human NM^18^, suggesting that at least partial AA synthesis may occur in this tissue. AA can then be stored in PLs and neutral glycerides or undergo further elongation/desaturation steps to generate adrenic acid (22:4n-6) and n-6 docosapentaenoic acid (22:5n-6). Upon release from PLs by phospholipases A2 (PLA2s), AA can be converted to various bioactive eicosanoids, depending on the activities of cyclooxygenases, lipoxygenases, or cytochrome P450 monoxygenases^19,20^. There is strong evidence that AA-derived eicosanoids are the central components of the proinflammatory processes. n-3 PUFAs EPA and DHA can also be metabolized by the same enzymes, resulting in compounds that have lower proinflammatory properties^21,22^. Hence, the local concentrations of these PUFAs ultimately govern the types and concentrations of eicosanoids that can be formed during inflammation processes. Higher concentrations of AA than those of EPA and DHA in human NM might contribute to perpetual inflammation in peripheral olfactory tissues of individuals exposed to environmental agents and chemicals. Positive association between nasal AA and hyposmia supports this hypothesis. However, the small number of hyposmic subjects included in the present study is a weakness and do not allow to draw firm conclusions. Similar analyses should be done in a larger number of patients with olfactory deficiency to confirm that a high level of nasal AA can be related to hyposmia.

LA and AA were found to be incorporated in the main PL classes, PC and PE, and in PI. In agreement with the PL structures observed in most tissues, these PUFAs were esterified at the sn-2 position of the glycerol moiety, while the sn-1 position was mainly occupied by SFAs (16:0 and 18:0). LA and AA can be released from PLs by a number of PLA2s, including secreted PLA2s, cytosolic Ca^2+^-dependent PLA2s, and Ca^2+^-independent PLA2s^23,24^. Several of these PLA2s have been detected in human NM; PLA2s expressed at the highest levels include PLA2G6 (a cytosolic Ca^2+^-independent PLA2), PLA2G12A (a secreted PLA2), and PLA2G16 (a lysosomal PLA2)^18^. These findings indicate that human NM has enzymatic machinery to liberate PUFAs from PLs. In the present study, we also demonstrated that PlsEs are an important subgroup of the PE class (28% of total PE species) in human NM and that the major PlsE species contained AA. AA-containing PlsCs were also detected. Plasmalogens constitute a special group of PLs characterized by the presence of fatty alcohols (mainly 16 and 18 carbon chains) with a vinyl-ether bond at the sn-1 position of the glycerol backbone^25^. Due to the vinyl ether bond, these compounds play a critical role in the cell membranes, providing unique structural attributes and protecting membrane lipids from oxidation^26^. These compounds were also suggested to function as reservoirs for lipid mediators involved in inflammatory responses^19,20^. The high AA content in the PlsE species and notable expression of PLA2G6, a PLA2 with preferential activity toward plasmalogens, suggest that this PL subgroup may play a crucial role in the control of AA availability in human NM.

In addition, we observed that NM from smokers had significant lower levels of 16:1n-7 and 16:1n-9 than those from nonsmokers (p = 0.030 and p = 0.022, respectively). The biological properties of these MUFAs are incompletely understood. In vivo and in vitro studies have shown that these compounds possess anti-inflammatory activity^27,28^. 16:1n-7 is also involved in other cellular functions, such as growth, proliferation and endoplasmic reticulum stress^29^. It is difficult to explain these deficiencies based on currently available information. Substances in cigarette smoke may impair the activity of stearoyl-CoA desaturase, the enzyme that catalyzes the introduction of a double bond in several saturated fatty acyl-CoAs^30^. Reduced stearoyl-CoA desaturase activity may result in lower levels of 16:1n-7 and 16:1n-9.

We did not detect an association between smoking and olfactory alterations in our study. This result may be related to small number of subjects, which could have impacted the statistical power of correlation analysis and/or to the fact that relatively high doses of cigarette smoke over a prolonged period of time are needed to produce olfactory dysfunction^31^. A number of studies have assessed whether smoking is a risk factor for poor olfaction and demonstrated a significant association with smoking; however, other studies reported no associations. Nevertheless, recent investigations (a meta-analysis and large population-based studies) have provided strong evidence for an association between chronic smoking and olfactory dysfunction^32–34^.

Notably, the amount of n-3 PUFAs (3–4% of total FAs) detected in human nasal samples was considerably lower than that detected in rodent olfactory mucosa^8^. This result suggests that nasal n-3 PUFA content might be species-specific. Nevertheless, this discrepancy can also be due to the nature of collected samples. In rodents, olfactory mucosa is clearly defined and can be harvested without contamination by respiratory tissue. In contrast, in humans, the boundaries between olfactory and respiratory mucosa are irregular, and patches of respiratory tissue can be interspersed in olfactory mucosa^11–13^. Although the biopsies were taken from an area rich in olfactory tissue, we cannot be certain that the samples contained only olfactory tissue. The characterization of the tissues in each biopsy could not be carried out because most of the samples were too small to perform both a comprehensive lipid analysis and a molecular RNA analysis. Nevertheless, RNA analyses performed on some biopsies indicated that respiratory genes were expressed in these samples. We therefore assume that the nasal samples collected in the present study may contain both olfactory and respiratory tissues. If this is the case, the lipid profiles were representative of both tissues, which makes it difficult to compare the lipid composition of human nasal samples with that of rodent olfactory mucosa.

Additionally, the present study analyzed the FA composition of nasal ceramides. Ceramides are a family of lipid molecules known to be involved in major cellular events, including growth, differentiation, and cell death. Ceramides are also precursors of complex lipids that play important structural and regulatory roles in the cells^35^. Ceramides consist of a sphingoid long-chain base linked to a FA via an amide bond. Glucose or galactose can be added to ceramide molecules, resulting in glucosylceramides or galactosylceramides (abbreviated as Hex-Cer in the present study). Subsequent attachment of a galactose molecule to glucosylceramides results in lactosylceramides (abbreviated as Hex2-Cer in the present study), which are precursors of complex glycosphingolipids, such as gangliosides. Ceramides can contain a wide spectrum of saturated or monounsaturated FAs, ranging from 14 to 26 carbon atoms in length. During de novo synthesis, acylation of the sphingoid long-chain base is carried out by a family of ceramide synthase enzymes (CerS). Each CerS has a high degree of FA specificity^36^. Some CerS isoforms are ubiquitously expressed, and other isoforms have a very specific distribution in tissues. A transcriptomic analysis performed on human NM indicated the presence of 6 CerS isoforms in this tissue, with CerS2, CerS4, and CerS5 being expressed at the highest levels^18^. These isoforms are responsible for the generation of Cer d18:1/16:0 and very long-chain ceramides Cer d18:1/24:1 and Cer d18:1/24:0^36^, the main species identified in human NM. In addition to de novo synthesis, ceramides can be generated by degradation of SM or by recycling glycosphingolipids (salvage pathway). Two sphingomyelinase isoforms, SMPD1 and SMPD4, and a ceramidase isoform (ASAH1) involved in the salvage pathway are expressed at a substantial level in human NM^18^, suggesting that these recycling pathways can contribute to the formation of nasal ceramides. The sphingomyelinase pathway was reported to enable rapid and transient production of ceramides, while the de novo pathway, which requires multiple enzymatic steps, is responsible for slow and robust accumulation of ceramides over a period of several hours^37^.

Ceramides and glycosphingolipids contribute to the structural organization and dynamic properties of the membranes^38^. Together with cholesterol and SM, glycosphingolipids form highly organized microdomains or “lipid rafts”, which are involved in various cellular functions, including cell–cell interactions and cell signaling^39^. Activation of sphingomyelinases in response to diverse stimuli induces the generation of ceramides within the rafts^40^. Then, ceramide molecules spontaneously associate to form ceramide-enriched membrane platforms that are involved in reorganization and clustering of proteins involved in intracellular signalosomes^40^. An increase in the ceramide levels can mediate many cell stress responses, including the regulation of cell growth, differentiation, senescence, proliferation, and cell death^37,41^. Ceramides and their phosphorylated derivatives are also implicated in inflammation^42^. These compounds can induce the expression or activation of the proinflammatory transcription factor NF-kappaB, which regulates the expression of many genes involved in inflammatory responses, such as cytokines and enzymes catalyzing the formation of prostaglandins^43^. The 16:0-ceramide species were shown to be mostly proapoptotic, whereas very long-chain 24- and 24:1-ceramides promote cellular proliferation^44–46^. Opposite effects of these ceramides contribute to the maintenance of cellular homeostasis. The role of ceramides detected in human NM remains to be explored.

Cholesterol would be also an important component of human sinus mucosa^10^. Several CEs, including cholesteryl linoleate and cholesteryl arachidonate, have been detected in this tissue^10^, indicating that LA and AA can be incorporated in this lipid group in addition to PLs. We did not examine CEs in the present study because our HPLC conditions (HILIC) were not adapted for analyzing these non-polar molecules. Likewise, our analytical conditions did not allow to analyze cardiolipins because these molecules have rather an apolar character. Moreover, gangliosides could not be characterized because their analysis by HPLC-HILIC/MS is influenced by ion suppression effects due to co-elution with more abundant PLs. Purification of gangliosides prior to HPLC-HILIC/MS analysis would enable to circumvent this problem^47^ but unfortunately, nasal samples did not contain enough biological material to carry out this purification step.

Like many tissues, NM is not easily accessible. To circumvent this issue, a number of studies have investigated relationships between circulating FAs and the FA composition of tissues, such as the brain and retina^48–50^. Blood fractions such as erythrocytes, plasma and PL or CE extracted from these components might be reliable biomarkers of tissue FA status but there is currently no scientific consensus regarding what fractions could be the most relevant biomarkers. The FA composition of plasma likely reflects the lipid status of other tissues and also represents the endogenous processing of the lipids and their recent dietary intake^51^. FA compositions in plasma CEs and PL are considered fairly independent of fasting status mainly because of their relatively slow turnover^52^. Similarly, the FA composition of erythrocytes is representative of long-term dietary intake because these cells have a life span of approximately 120 days^53^. Therefore, in the present study, we assessed the correlations between various circulating FA fractions and the FA nasal content. Moderate positive association between the AA content in the nasal tissue and that in the plasma is one of the major results of our study. Interestingly, significant correlations were observed in the case of total plasma and the plasma CE and PL fractions. Our results are in agreement with the data of a previous study that demonstrated a strong correlation between FA proportions in CEs and PLs in various Swedish populations, suggesting that these two plasma fractions can be used as interchangeable biomarkers^54^. In the present study, similarities between total plasma and plasma fractions (CE and PL) may be explained by the fact that FA analyses were performed in the fasting plasma samples. Notably, a quite strong association was observed in the case of AA esterified in plasma-CE (*r* = 0.68), suggesting that this marker can be a good indicator of nasal AA levels. This observation is consistent with a recent study showing that plasma CEs might be relevant biomarkers of retina PUFAs^55^. Taken together, these results indicate that the PUFA content of plasma-CE might reflect that of neuronal tissues such as the NM and retina. Our finding also suggests that plasma lipoproteins (such as LDL and HDL) that contain AA esterified within CE or PL may supply AA to NM. Correlations between the plasma AA level and nasal AA level suggest an efficient uptake of AA from the circulating lipid pools by NM cells.

In conclusion, the present study showed that human NM is characterized by a high content of n-6 PUFA species, which might have repercussions on olfactory function, as suggested by significant associations between nasal AA (20:4n-6) levels and olfactory deficiency. Multiple ceramide species involved in cell death and proliferation were also detected. Our findings suggest that these lipid compounds may be involved in the control of NM homeostasis. Identification of plasma CE as a circulating biomarker might be useful for prediction of nasal AA levels.

## Material and methods

### Patients

The study was performed according to the guidelines of the Declaration of Helsinki and has been formally approved by the Dresden Hospital Ethics Committee (ethics protocol number EK118032015). Twenty-three patients (9 females and 14 males) who underwent septoplasty or other nasal operations under general anesthesia were recruited. All subjects gave written informed consent to participate in the study. Various clinical characteristics, such as sex, age, body mass index and smoking status, were recorded (Table 1). Before surgery, patients were tested for odor identification by a validated psychophysical olfactory test using 12 different odors, which is known as the ‘‘Sniffin’ Sticks’’ test^56^. The cutoff score between normosmia and hyposmia is the 10th percentile of the normal distribution of results obtained in the odor identification test^57^.

### Biopsies

Nasal tissue samples were acquired from the upper portions of the middle turbinate and the uncinate process because olfactory mucosa was shown to be the predominant tissue in these nasal regions^58,59^. Biopsies were collected from only one side of the nasal cavity. Blood was sampled by venipuncture after fasting and collected in heparinized tubes; then, erythrocytes were separated from the plasma by centrifugation at 3,000 rpm for 10 min at + 4 °C. Erythrocytes were washed three times with an isotonic saline solution. All samples were immediately stored at − 80 °C until further analyses.

### Lipid analyses

#### Chemicals and lipid standards

Chloroform (CHCl_3_), methanol (CH_3_OH), ammonium acetate, acetonitrile (ACN), and H_2_O of HPLC–MS grade were purchased from Fisher Scientific (Illkirch, France). Commercially available PL and sphingolipid standards were purchased from Avanti Polar Lipids INC-Coger (Paris, France). Other chemical reagents were obtained from Merck KGaA (Darmstadt, Germany). PL class standards (egg PG, liver PI, brain PE, brain PS, brain PC and brain SM) were used to quantify PL classes in nasal samples. Internal PL standards composed of PC(14:0/14:0), PC(24:0/24:0) and PE(14:0/14:0) were added for the quantification of PL molecular species. PC(18:0/18:0), PC(18:0/20:4), PE(18:0/18:1), PE(18:1/18:1), PlsE(18:0p/18:1), PlsE(18:0p/20:4), PlsE(18:0p/22:6), LPE(16:0), LPC(16:0), PI(18:1/18:1), PS(16:0/18:1), brain Cer, glucosylCer(d18:1/16:0), glucosylCer(d18:1/18:0), galactosylCer(d18:1/16:0), galactosylCer(d18:1/18:0), galactosylCer(d18:1/24:0), lactosylCer(d18:1/24:1) were used for the optimization of analytical methods. All lipids were purchased from Avanti Polar lipids, INC-Coger (Paris, France).

#### Lipid extraction from the plasma, erythrocytes, and nasal mucosa

Total lipids were extracted from the plasma and erythrocytes according to the method of Moilanen et al.^60^. Briefly, 10 mL of CHCl_3_/CH_3_OH (1/1, v/v) was added to 1 mL of sample. After shaking and centrifugation (1500*g*, 3 min), 3 mL of CHCl_3_ and 1.8 mL of a solution containing 17 mM NaCl and 1 mM H_2_SO_4_ were added to the liquid phase. After vigorous shaking, the mixture was centrifuged (1500*g*, 3 min), and the lower phase was collected and evaporated under a stream of nitrogen. Total lipids were redissolved in CHCl_3_/CH_3_OH (1:1, v/v) and stored under nitrogen at − 30 °C until further analyses.

Total lipids from NM were extracted according to the Folch method^61^. Briefly, each sample was homogenized in 2 mL of 0.73% NaCl using a tissue lyser (Qiagen, Venlo, Netherlands, 2 cycles of 7 min at 30 Hz). Total lipids were then extracted with 10 mL of CHCl_3_/CH_3_OH (2:1, v/v). The mixture was centrifuged (1500*g*, 3 min), and the lower organic phase was collected and evaporated under a stream of nitrogen. Total lipids were redissolved in CHCl_3_/CH_3_OH 1:1 (v/v) and stored under nitrogen at − 30 °C until further analyses.

#### Preparation of plasma phospholipids and cholesteryl esters

PLs and CEs were separated from the lipid extracts of total plasma by thin-layer chromatography (TLC) as described previously^49^. Briefly, total lipids were separated by TLC on precoated silica gel plates (model TLC Silica gel 60, 20 cm × 20 cm, Merck, Darmstadt, Germany) using a solvent mixture of hexane/diethyl ether/acetic acid (80:20:1, v/v/v). After spraying with 2’,7’-dichlorofluorescein, the fluorescence signal of the silica gel plates was visualized under UV light (360 nm), and the spots corresponding to PLs and CEs were scraped off the plate. The silica scrapped off the TLC plate was then submitted to the transesterification procedure and analyzed by gas chromatography coupled to flame ionization detection (GC-FID) following the FA analysis procedure.

#### Fatty acid composition

Lipid extracts were evaporated to dryness under nitrogen. Then 0.7 ml of boron trifluoride-methanol (7%) and 0.3 ml of toluene were added to 1–20 mg of lipid. The tube was closed with the screw cap, and then heated at 95 °C using an oven for two hours, cooled and opened. After that, 5 ml of NaHCO_3_ solution and 5 ml of hexane were added. The mixture was shaken, centrifuged (1500*g*, 3 min) and the FA methyl esters were extracted from the upper phase (hexane). After transmethylation, FA methyl esters were analyzed by GC-FID as described previously^8^. Briefly, GC was performed on a Hewlett Packard model 5890 gas chromatograph (Palo Alto, CA) equipped with a CP-Sil 88 column (Varian, Les Ulis, France) and a flame ionization detector. Hydrogen was used as the carrier gas. FA methyl esters were identified by comparison with commercial and synthetic standards. The data were processed using EZChrom Elite software (Agilent Technologies, Massy, France) and reported as the percentage of total FAs. FA analyses were performed on 23 blood samples but only on 20 nasal samples because some nasal biopsies did not contain enough biological material to carry out this analysis.

#### Plasma cholesterol measurement

Total cholesterol was extracted from the plasma samples and quantified using the method described by Bretillon et al.^62^. Briefly, total lipids extracted from 50 µL of the plasma were subjected to alkaline hydrolysis in 1 M KOH (5 mL) for 2 h. After the addition of phosphoric acid (65 µL), the sterols were extracted with CHCl_3_ (9 mL) in the presence of 0.9% sodium chloride (3 mL). The organic phase was evaporated, and 80 µg of 5α-cholestane was added as an internal standard. Sterols were derivatized to trimethylsilyl ethers by heating at 60 °C in the presence of pyridine (200 µL) and *N,O*-bis(trimethylsilyl)trifluoroacetamide (200 µL), and then analyzed by GC-FID (Hewlett-Packard HP4890A) using conditions described previously in detail^62^. The data were processed using EZChrom Elite software (Agilent Technologies) and reported as mg cholesterol/mL plasma.

#### Separation and quantification of nasal phospholipid classes by liquid chromatography coupled to charged aerosol detector (Corona-CAD)

The phosphorus content of the total lipid extracts from nasal samples was determined according to the method developed by Bartlett and Lewis^63^ as described previously^64^. After dilution to a concentration of 500 µg/mL in CHCl_3_/CH_3_OH 1:1 (v/v), PL classes were separated by LC under the HILIC conditions and detected using a Corona™ ultra RS™ charged aerosol detector (CAD, Thermo Scientific, USA. Separation of PL classes was based on their increasing polarities (supplementary Fig. S3). PL classes were determined relative to total PLs in nasal sample. Analysis methods, including detector parameters, instruments, elution gradient, and other chromatographic separation conditions, were described in detail by Le Bon et al.^8^. PL classes were analyzed in only 19 nasal biopsies because some samples did not contain enough biological material to perform this analysis.

#### Structural analysis of nasal phospholipids by liquid chromatography coupled to mass spectrometry

PL classes were separated from lipid extracts by HPLC, under HILIC conditions, according to their increasing polarities. PL molecular species were characterized in the elution zone corresponding to each PL class, using a high-resolution MS. Then, the characterized species were quantified by targeted analyses using a triple quadrupole MS (see the workflow in supplementary Fig. S2). The analytical conditions of these methods were as follows.

##### Chromatographic separation of phospholipids

HPLC separation was performed using an UltiMate™ 3000 LC pump equipped with a dual-gradient pump and an UltiMate™ 3000 autosampler from Thermo Scientific (USA). The injection volume was 10 µL. PL classes were separated by LC under the HILIC conditions as described previously^8^ (supplementary Fig. S3). The HILIC conditions were similar to those used for the separation of PL classes when coupling the chromatographic system to Corona. The flow from LC was split using an analytical fixed flow splitter (split ratio = 1:1, postcolumn) from Analytical Scientific Instruments (El Sobrante, CA, USA). The LC system was controlled by Standard Instrument Integration software based on Dionex Chromeleon TN 7. Lipid extracts were diluted in CHCl3/CH3OH (1:1, v/v) and the concentration of the injected lipids for characterization purposes was 0.5 mg mL^-1^.

##### Characterization of phospholipid species

An Orbitrap Fusion™ Tribrid mass spectrometer (Thermo Scientific, USA) was used for high-resolution analyses of PL species in nasal tissues. The instrument was equipped with an EASY-Max NG™ ion source (heated electrospray ionization (H-ESI)). H-ESI source parameters were optimized and set as follows: ion transfer tube temperature of 285 °C, sheath gas flow rate of 35 a.u., auxiliary gas flow rate of 25 a.u., sweep gas flow rate of 1 a.u., and vaporizer temperature of 370 °C. Positive and negative ions were monitored alternatively by switching the polarity with a spray voltage set to 3500 V in the positive and negative ion modes. An Orbitrap mass analyzer was used to obtain all mass spectra in the full scan mode within a normal mass range and a target resolution of 120,000 (full-width at half maximum (FWHM) at *m/z* 200). All MS data were recorded using a maximum injection time of 50 ms, automatic gain control at 4.10^5^, and one microscan. An intensity threshold filter of 1.10^3^ counts was applied. For tandem mass spectrometry (MS/MS) analyses, data-dependent mode was used to characterize the PL species. Precursor isolation was performed in a quadrupole analyzer with an isolation width of *m/z* 1.6. Higher-energy collisional dissociation was used for fragmentation of PL species with an optimized stepped collision energy of 30% (± 5%). Linear ion trap was used to acquire spectra of the fragment ions in the data-dependent mode. The automatic gain control target was set to 2.10^4^ with a maximum injection time of 50 ms. All MS and MS/MS data were acquired in the profile mode. Orbitrap mass spectrometer was controlled by Xcalibur™ 4.1 software. All PL species were identified using high-accuracy data based on the information collected from the fragmentation spectra by LipidSearch™ software and the LIPID MAPS™ database (supplementary Fig. S3).

##### Quantification of phospholipid species

Samples were diluted to a concentration of 25 µg mL^−1^ PLs in CHCl_3_/CH_3_OH (1:1, v/v) for analysis. The LC system under the previously described HILIC conditions was coupled to a triple quadrupole mass spectrometer (Thermo Finnigan TSQ Quantum) equipped with a standard electrospray ionization source to quantify PL molecular species. Specific acquisition methods in the positive and negative ion modes were optimized and used according to the studied compounds (supplementary Fig. S3). PC, PlsC and SM species were quantified in the positive ion mode by precursor ion scanning of *m/z* 184 amu, which corresponds to the choline head group. PE species lose their ethanolaminephosphate head group as a neutral fragment of 141 Da. Therefore, neutral loss scanning of 141 Da in the positive ion mode was used for specific detection and quantification of PE. Similarly, PS species lose their serine-phosphate head group as a neutral fragment of 185 Da. Therefore, neutral loss scanning of 185 Da in the positive ion mode was used to quantify PS species. In the negative ion mode, PI species show a fragment at *m/z* 241 amu identified as inositolphosphate minus one molecule of H_2_O. This fragment was used for precursor ion scanning to quantify these compounds. The quantification of PlsE was performed in the negative ionization by multiple reaction monitoring mode (MRM) of the parent/fragment transition for each selected plasmalogen. The amount of PL species in each PL class was expressed relative to the total lipids in this class. The data were processed using Xcalibur software. In addition, isotopic overlap corrections were applied to the data^65^. Additional details about the electrospray source parameters and methods of mass spectrometry are available in Le Bon et al.^8^.

#### Analysis of nasal ceramides, hexosyl-ceramides, and dihexosyl-ceramides by liquid chromatography coupled to mass spectrometry

Cer, HexCer, and Hex2Cer classes were separated from lipid extracts by HPLC, under modified HILIC conditions, according to their increasing polarities. The amounts of Cer, HexCer, and Hex2Cer molecular species was determined using a high-resolution MS (see the workflow in supplementary Fig. S2). The analytical conditions of these methods were as follows.

##### Chromatographic separation

HPLC separation of the Cer, HexCer, and Hex2Cer classes was performed using a Dionex UltiMate™ 3000 LC pump from Thermo Scientific equipped with an UltiMate™ 3000 autosampler. Knowing that Cer, HexCer, and Hex2Cer are less polar than PL, the separation of these sphingolipid classes was achieved under adapted parameters of the HILIC method already used to analyze PL classes using an Accucore™ HILIC LC column (150 × 2.1 mm, 2.6 µm). The injection volume was 5 µL, and the column was maintained at 40 °C. The new mobile phase consisted of (A) ACN/H_2_O (99:1, v/v) containing 10 mM ammonium acetate and (B) ACN/H_2_O (50:50, v/v) containing 10 mM ammonium acetate. The solvent-gradient system of the analytical pump was as follows: 0 min 100% A, 10 min 92% A, 40 min 50% A, and 41–60 min 100% A. A flow rate was 500 µL min^−1^. Lipid extracts were diluted in CHCl3/CH3OH (1:1, v/v) and the concentration of the injected lipids was 0.5 mg mL^−1^.

##### Characterization and quantification of Cer, HexCer, and Hex2Cer species

An Orbitrap Fusion™ Tribrid mass spectrometer equipped with an H-ESI source was used for the analysis of Cer, HexCer, and Hex2Cer molecular species from nasal samples. Identification of these compounds is based on their retention time using lipid standards, on the data acquired at a high resolution, and on tandem MS experiments. The retention time of lipid standards on the total ion current chromatogram enabled identification of elution zones for each sphingolipid class (supplementary Fig. S3). High-resolution full scan experiments were used to acquire exact masses of analyzed species, and information on FAs and long-chain bases (sphingosines) of Cer, HexCer, and Hex2Cer was obtained from the MS/MS spectra. Data processing of high-resolution MS and MS/MS experiments was performed by Xcalibur™ software with the help of LipidSearch™ software and the LIPID MAPS™ database. Additional details of the source parameters and the MS and MS/MS methods used for identification are described in the section “Characterization of phospholipid species”.

The quantitative analysis of Cer, HexCer, and Hex2Cer molecular species within a specific sphingolipid class was performed using the high-resolution full scan data acquired by the Orbitrap Fusion mass spectrometer in the positive ion mode. Sphingolipids were detected as protonated ions [M + H]^+^ by positive ionization. The proportion of individual species of a specific (Cer, HexCer, or Hex2Cer) class was calculated as the ratio of its MS signal to the sum of all detected peak signals in this class.

### Statistical analyses

The Spearman correlation coefficients were calculated to determine associations between nasal and blood FA profiles using XLSTAT v2019.1.2 (build 57072) software (Addinsoft). The relationships between nasal FA levels and individual characteristics were analyzed using R software (version 3.5.2)^66^. The Spearman correlation test was used for continue variables (stat_cor, *ggpubr* package), and the Wilcoxon test was used to compare the mean values of the discrete variables (stat_compare_means, *ggpubr* package). Type II ANCOVA (glm, *stats* package) was used to test the influence of various factors (sex, smoking status, and olfactory diagnosis) and covariates (age and BMI) on FA levels and to calculate the p-values for each main effect corrected for all other effects. For all data analyses, the correlations were considered significant at p < 0.05. Schober’s classification thresholds^67^ were followed to interpret the correlation coefficients: 0.10 to 0.39, weak; 0.4 to 0.69, moderate; 0.70 to 0.89, strong; and ≥ 0.9, very strong.

## Supplementary Information

Supplementary Figure S1.

Supplementary Figure S2.

Supplementary Figure S3.

Supplementary Table S1.

Supplementary Table S2.

## Data Availability

Raw data are available on reasonable request to the corresponding author A.M.L.B.
