# Supplementary material for: Lipidomic profile of human nasal mucosa and associations with circulating fatty acids and olfactory deficiency
Source: Sci Rep. 2021 Aug 18;11:16771. doi: 10.1038/s41598-021-93817-1 (PMC8373950; doi:10.1038/s41598-021-93817-1)

Lipidomic profile of human nasal mucosa and associations with circulating fatty acids and olfactory deficiency

Spiro Khoury[1], Volker Gudziol[2], Stéphane Grégoire[1], Stéphanie Cabaret[1], Susanne Menzel[2], Lucy Martine[1], Esther Mezière[1], Vanessa Soubeyre[1], Thierry Thomas-Danguin[1], Xavier Grosmaitre[1], Lionel Bretillon[1], Olivier Berdeaux[1], Niyazi Acar[1], Thomas Hummel[2], Anne Marie Le Bon[1*]

[1] Centre des Sciences du Goût et de l'Alimentation, AgroSup Dijon, CNRS, INRAE, Université Bourgogne Franche-Comté, F-21000 Dijon, France.
[2] Department of Otorhinolaryngology, Interdisciplinary Center Smell and Taste, TU Dresden, Dresden, Germany.

Supplementary Figure 1

Supplementary Figure S1: Associations between nasal fatty acids and individual characteristics (n=20). The relationships were analyzed using the Wilcoxon test (smoking status, olfactory diagnosis, and sex) or the Spearman test (BMI and age).

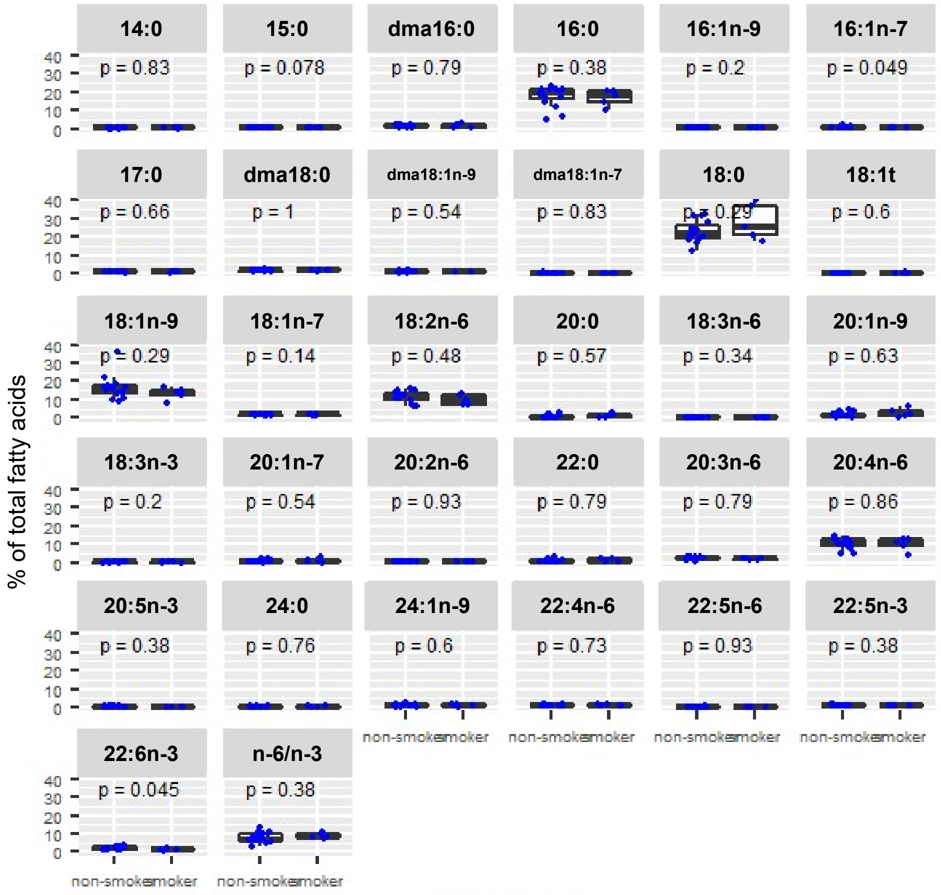

Smoking status

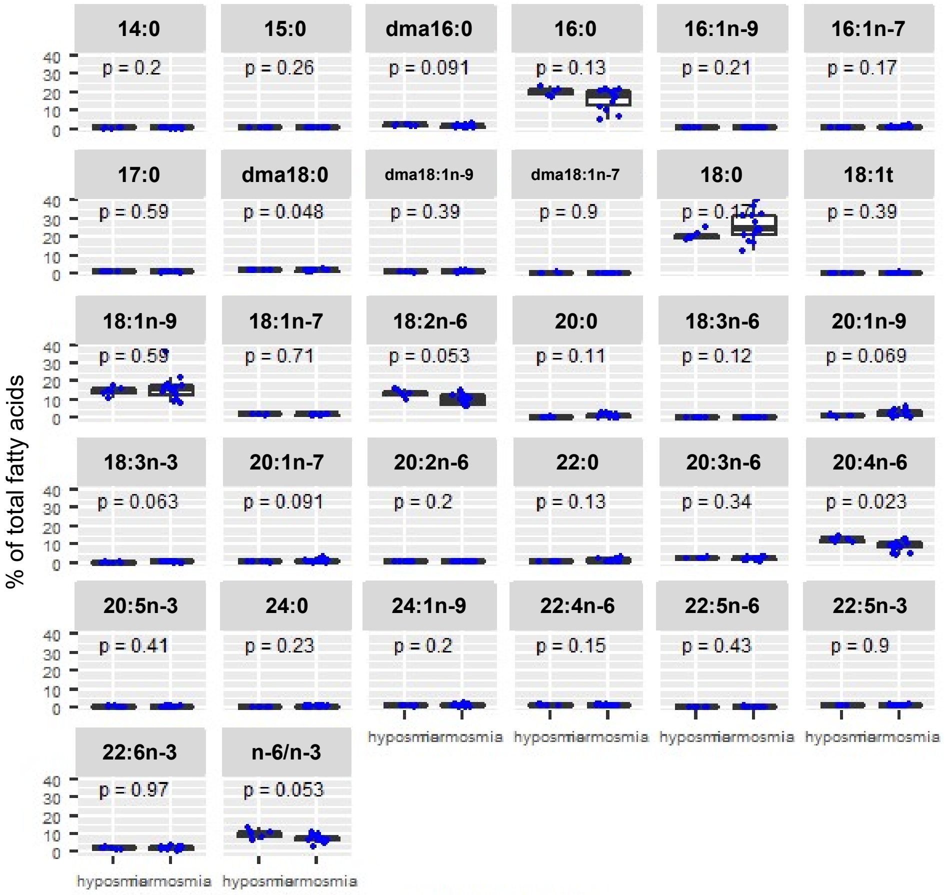

Olfactory diagnosis

Supplementary Figure S1 (continued)

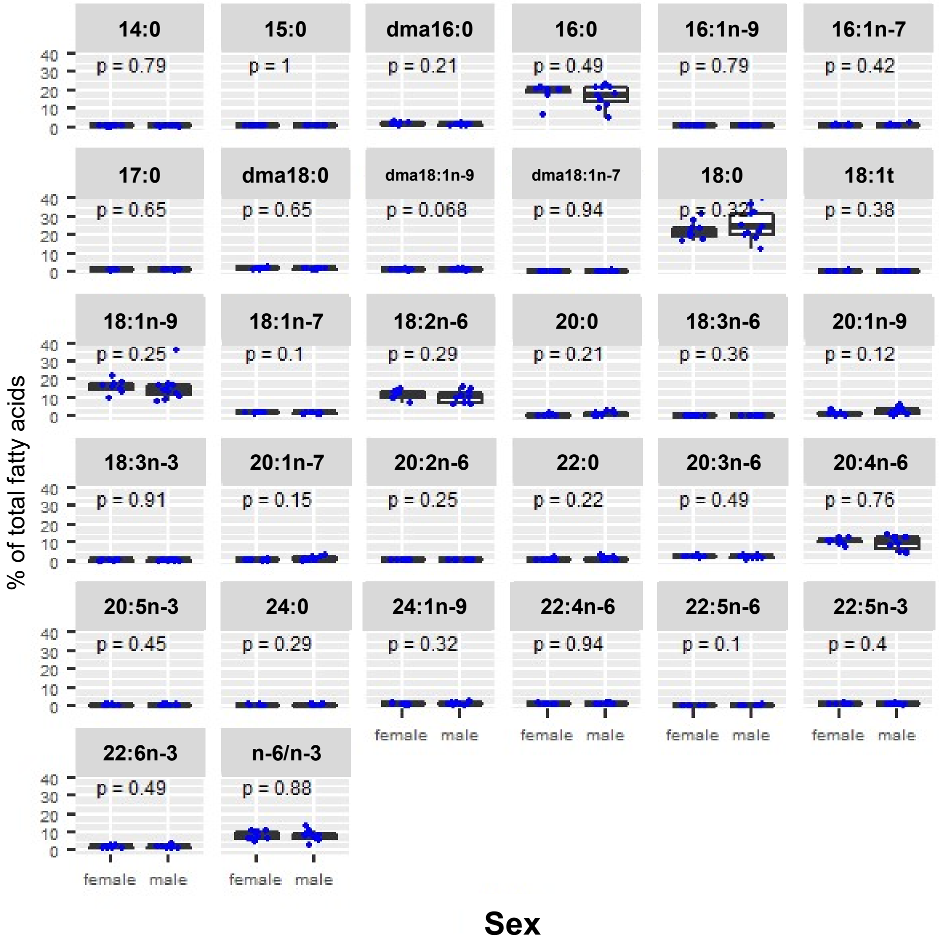

Supplementary Figure S1 (continued)

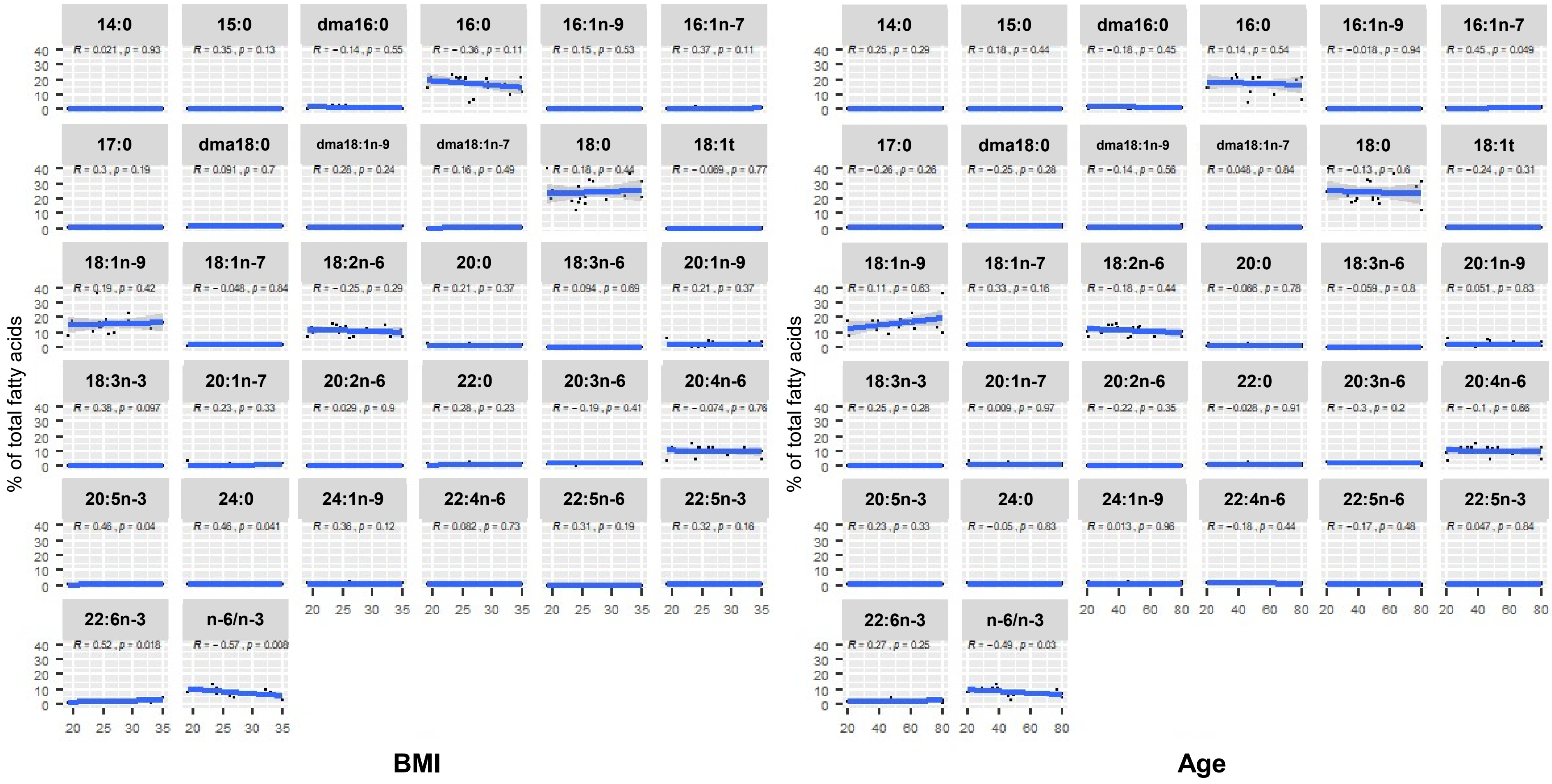

Supplement: Supplementary file 1 — Supplementary Figure S1. [file 41598_2021_93817_MOESM1_ESM.pdf]
