# Supplementary material for: Lipidomic profile of human nasal mucosa and associations with circulating fatty acids and olfactory deficiency
Source: Sci Rep. 2021 Aug 18;11:16771. doi: 10.1038/s41598-021-93817-1 (PMC8373950; doi:10.1038/s41598-021-93817-1)
Supplement: Supplementary file 2 — Supplementary Figure S2. [file 41598_2021_93817_MOESM2_ESM.pdf]

Lipidomic profile of human nasal mucosa and associations with circulating fatty acids and olfactory deficiency

Spiro Khoury[1], Volker Gudziol[2], Stéphane Grégoire[1], Stéphanie Cabaret[1], Susanne Menzel[2], Lucy Martine[1], Esther Mezière[1], Vanessa Soubeyre[1], Thierry Thomas-Danguin[1], Xavier Grosmaitre[1], Lionel Bretillon[1], Olivier Berdeaux[1], Niyazi Acar[1], Thomas Hummel[2], Anne Marie Le Bon[1*]

[1] Centre des Sciences du Goût et de l'Alimentation, AgroSup Dijon, CNRS, INRAE, Université Bourgogne Franche-Comté, F-21000 Dijon, France.
[2] Department of Otorhinolaryngology, Interdisciplinary Center Smell and Taste, TU Dresden, Dresden, Germany.

Supplementary Figure 2

Supplementary Figure S2: Workflow illustrating the different analytical methods used in this study to analyze fatty acids, phospholipid classes, phospholipid molecular species, and ceramide molecular species in human nasal mucosa.

The number of analyzed samples (n) varied from 19 to 23 because in some biopsies, the amount of collected nasal tissues was not sufficient to carry out all scheduled analyses.

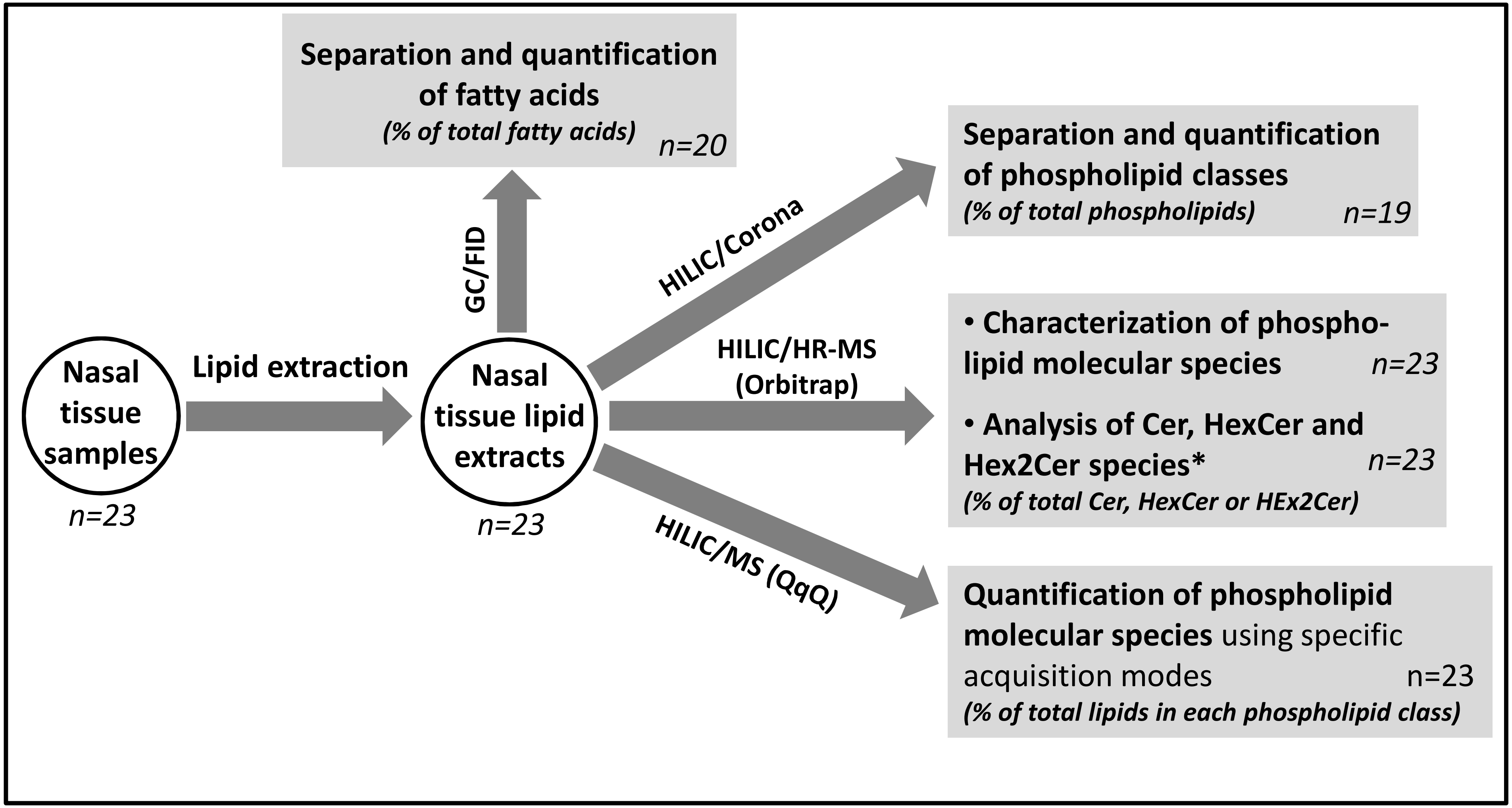

* Cer: ceramides, HexCer: hexosyl-ceramides, Hex2Cer: dihexosyl-ceramides.
