# Supplementary material for: Lipidomic profile of human nasal mucosa and associations with circulating fatty acids and olfactory deficiency
Source: Sci Rep. 2021 Aug 18;11:16771. doi: 10.1038/s41598-021-93817-1 (PMC8373950; doi:10.1038/s41598-021-93817-1)
Supplement: Supplementary file 3 — Supplementary Figure S3. [file 41598_2021_93817_MOESM3_ESM.pdf]

Lipidomic profile of human nasal mucosa and associations with circulating fatty acids and olfactory deficiency

Spiro Khoury[1], Volker Gudziol[2], Stéphane Grégoire[1], Stéphanie Cabaret[1], Susanne Menzel[2], Lucy Martine[1], Esther Mezière[1], Vanessa Soubeyre[1], Thierry Thomas-Danguin[1], Xavier Grosmaitre[1], Lionel Bretillon[1], Olivier Berdeaux[1], Niyazi Acar[1], Thomas Hummel[2], Anne Marie Le Bon[1*]

[1] Centre des Sciences du Goût et de l'Alimentation, AgroSup Dijon, CNRS, INRAE, Université Bourgogne Franche-Comté, F-21000 Dijon, France.
[2] Department of Otorhinolaryngology, Interdisciplinary Center Smell and Taste, TU Dresden, Dresden, Germany.

Supplementary Figure 3

Supplementary Figure S3: Chromatograms and mass spectra representative of lipid molecules analyzed in human nasal mucosa.

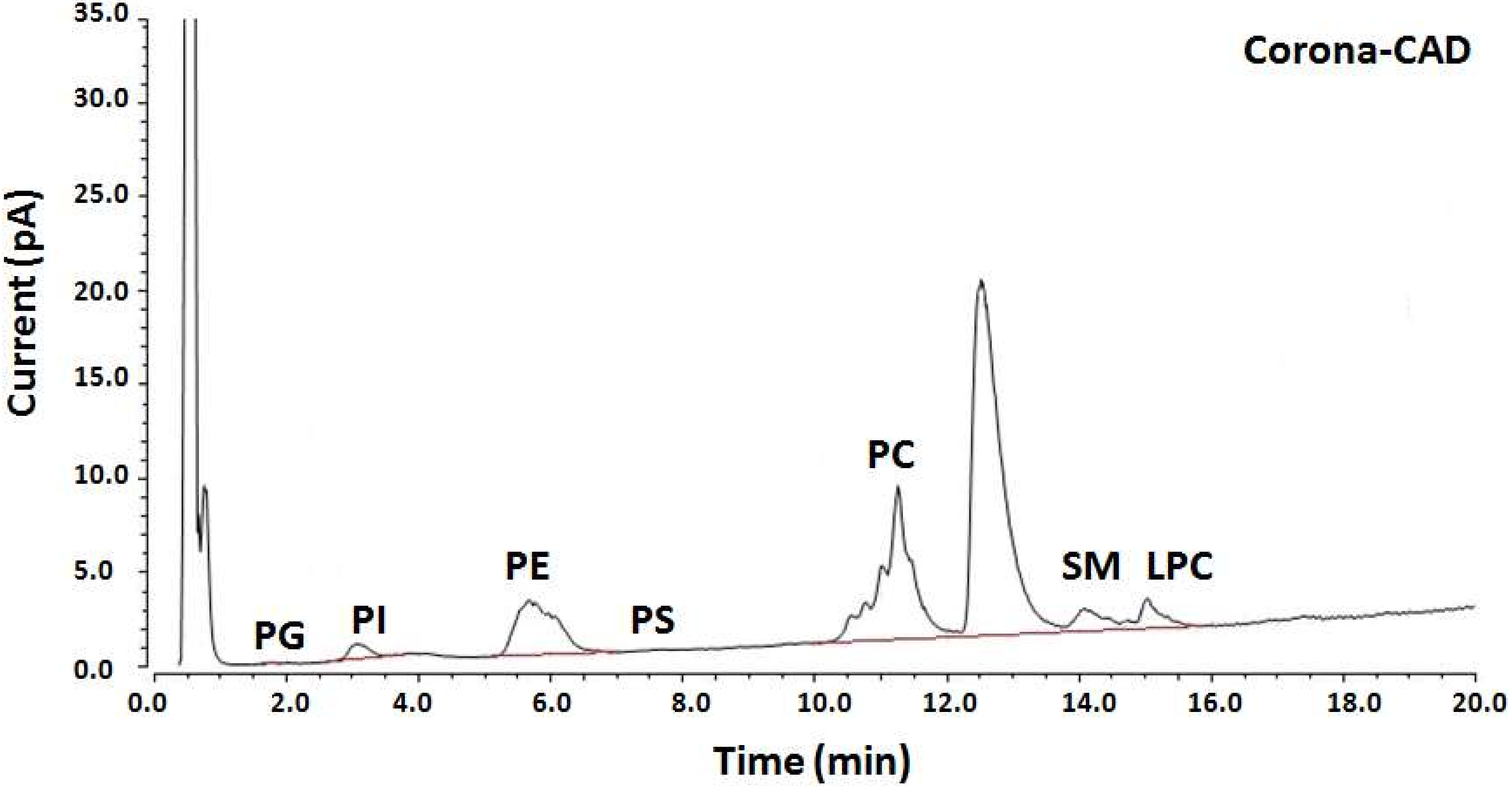

Separation of the different phospholipid classes from a nasal mucosa sample by HILIC/Corona-CAD, after lipid extraction by the Folch method,

Supplementary Figure S3 (continued)

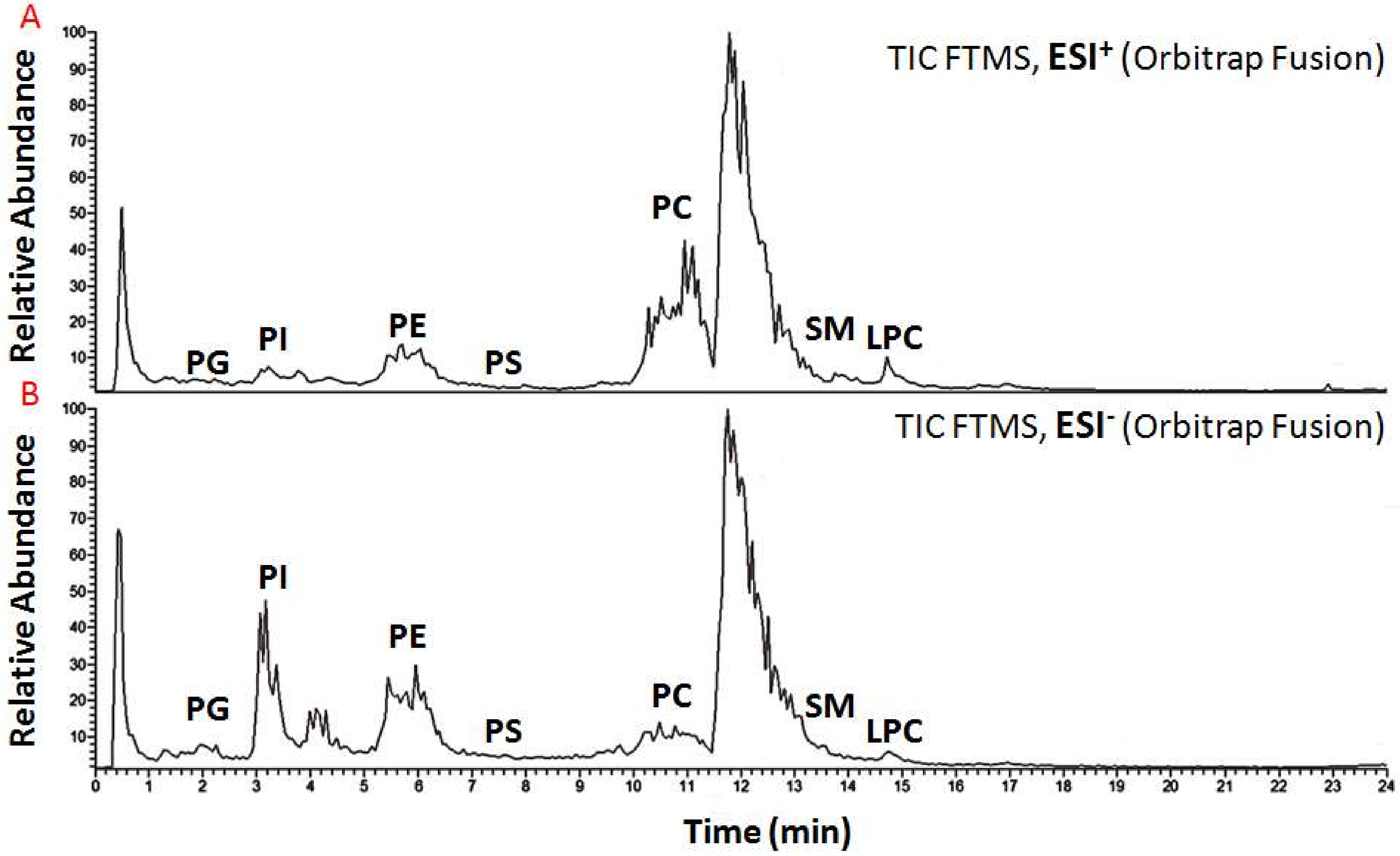

Total Ion Chromatogram (TIC) showing the separation of the different PL classes from a nasal mucosa sample after lipid extraction by the Folch method. Analysis of lipid extract was performed by HILIC/ESI HR-MS (Orbitrap Fusion) in positive (A) and negative (B) ionization.

Supplementary Figure S3 (continued)

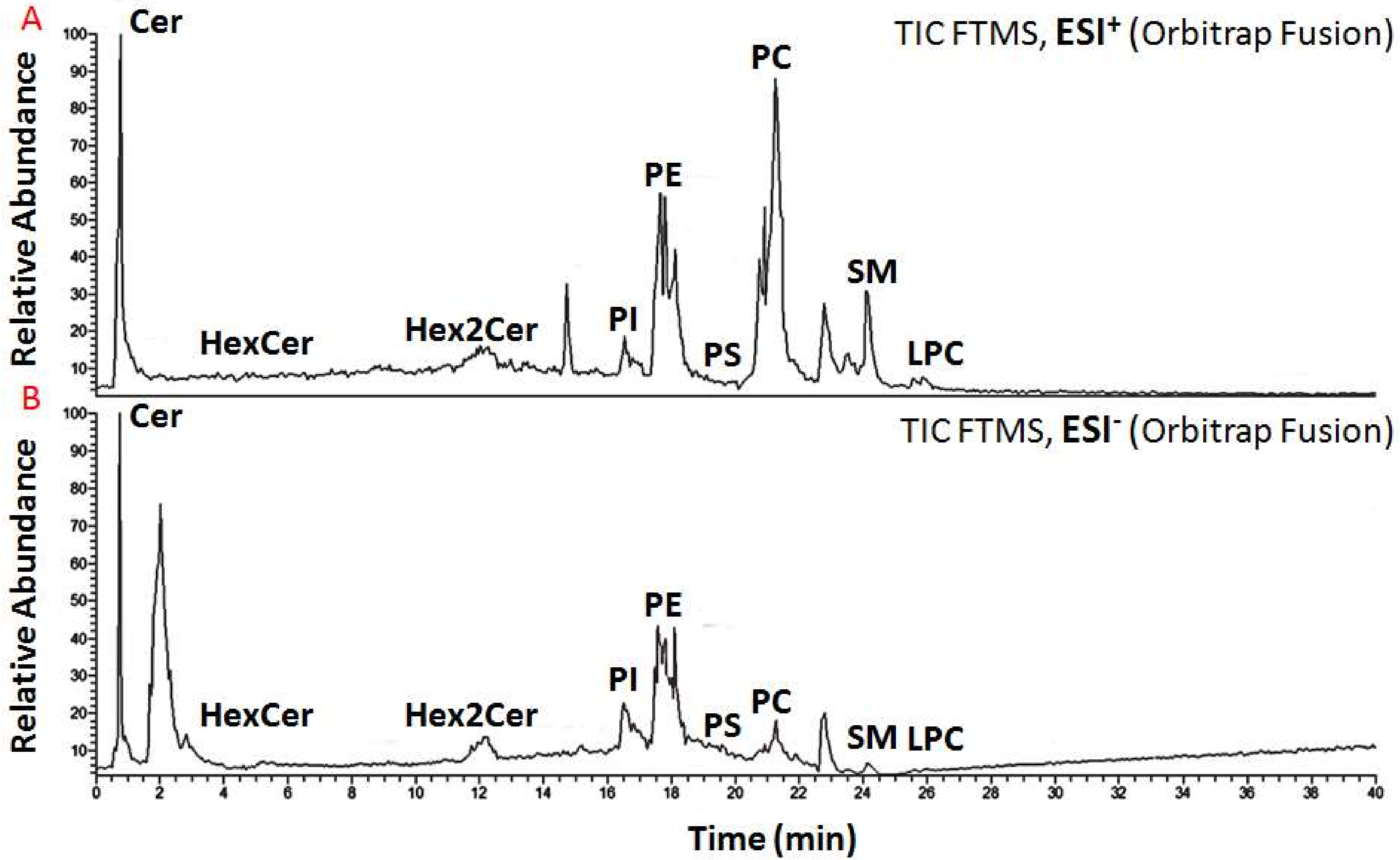

Total Ion Chromatogram (TIC) showing the separation of Cer, HexCer, Hex2Cer and the different PL classes from a nasal mucosa sample after lipid extraction by the Folch method. Analysis of lipid extract was performed by the modified HILIC method coupled with ESI HR-MS (Orbitrap Fusion) in positive (A) and negative (B) ionization.

Supplementary Figure S3 (continued)

MS/MS spectra of PC(16:0/18:1) obtained using the Orbitrap Fusion in positive ion mode (A) and negative ion mode (B), showing the different fragment ions characteristic for this species. The spectrum in ESI$^+$ allowed the identification of the polar head of PC class (characteristic fragment ion with m/z=184). The spectrum in ESI$^-$ allowed the identification of FA alkyl chains (FA16:0 m/z=255 and FA18:1 m/z=281) and other fragment ions.

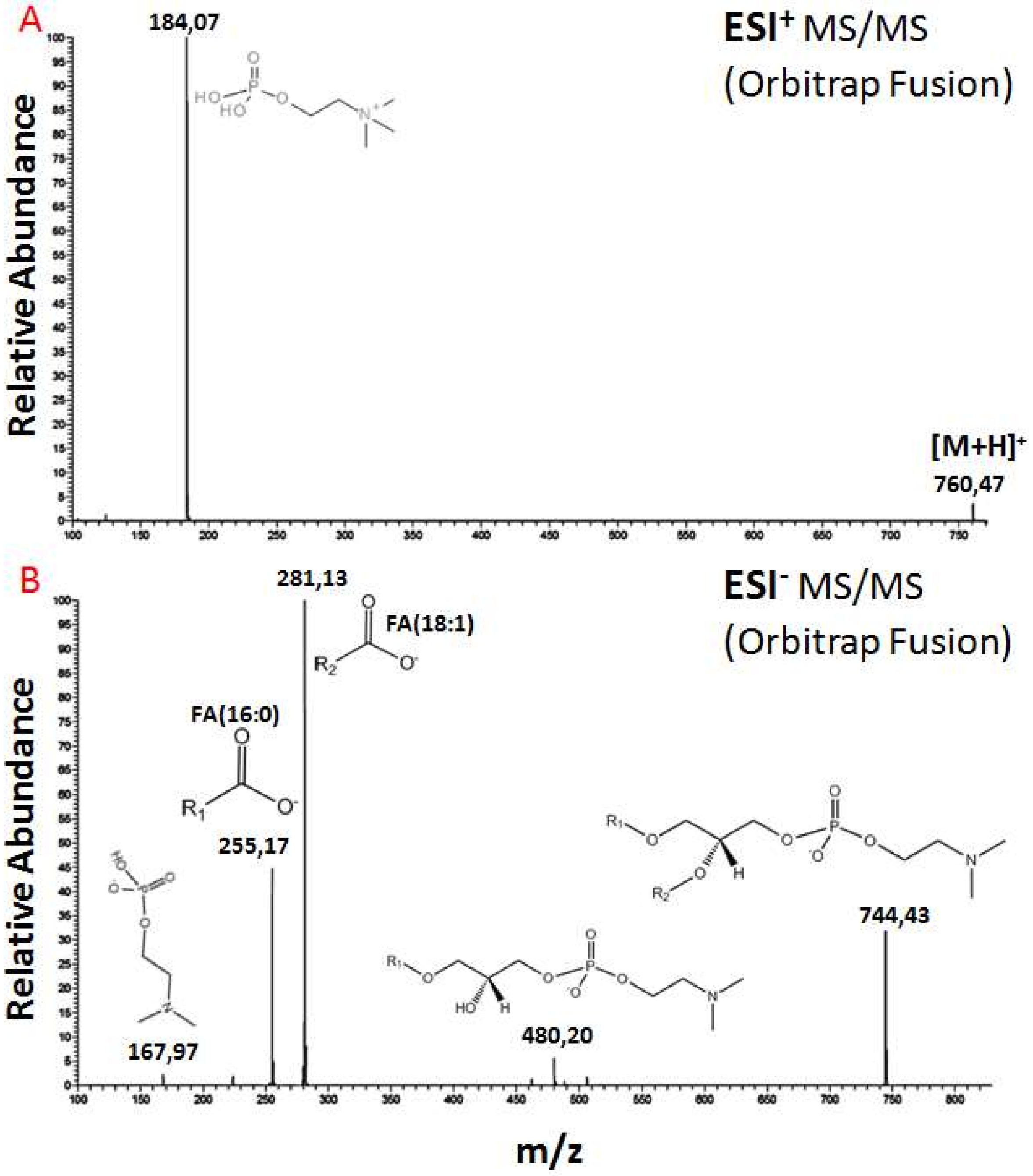

Supplementary Figure S3 (continued)

MS/MS spectra of PE(18:1/22:6) obtained using the Orbitrap Fusion in positive ion mode (A) and negative ion mode (B), showing the different fragment ions characteristic for this species. The spectrum in ESI$^+$ showed a major ion corresponding to the neutral loss of 141 Da from $[M+H]^+$ ion. The spectrum in ESI$^-$ allowed the identification of FA alkyl chains (FA18:1 m/z=281 and FA22:6 m/z=327) and other fragment ions

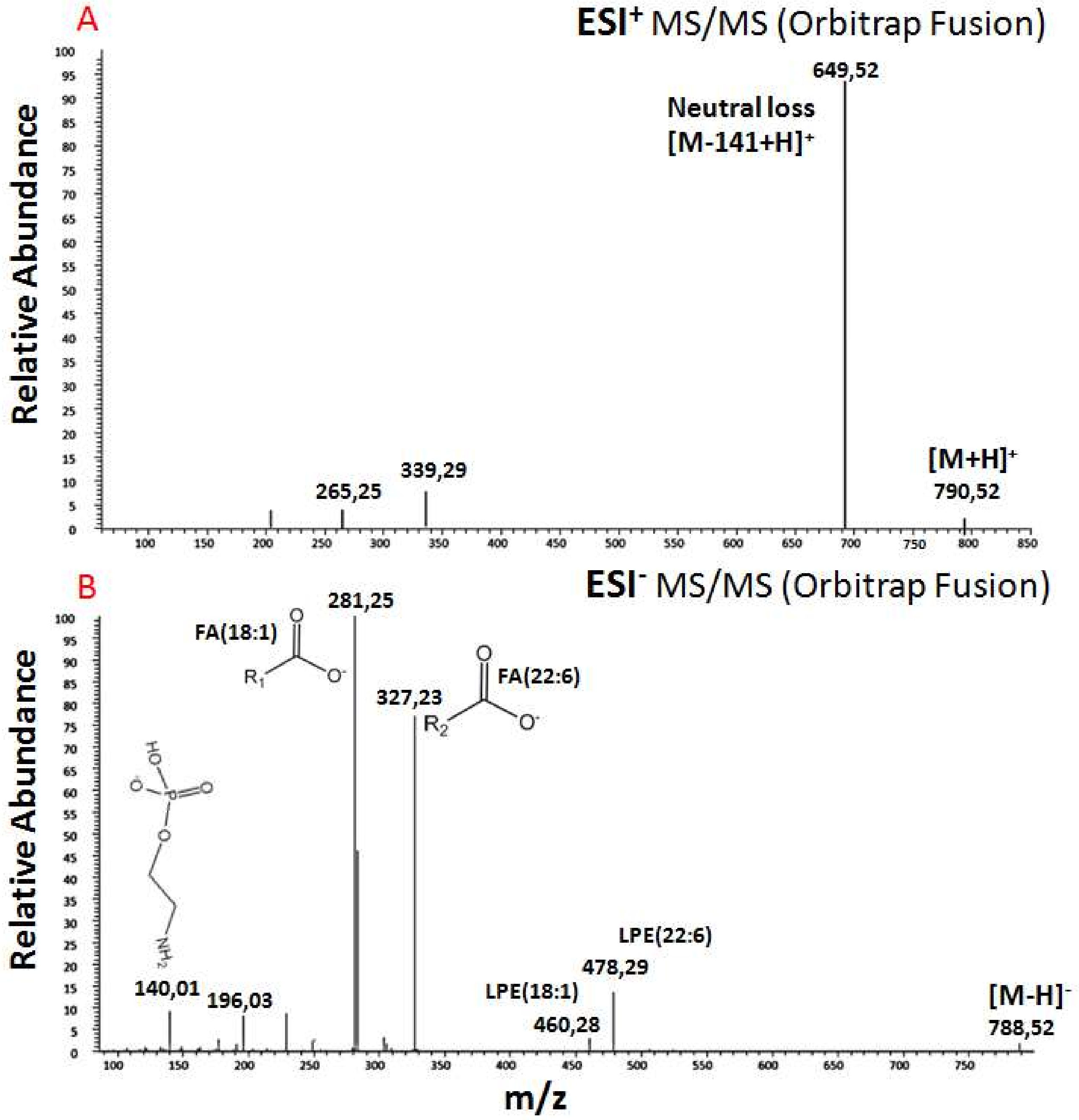

Supplementary Figure S3 (continued)

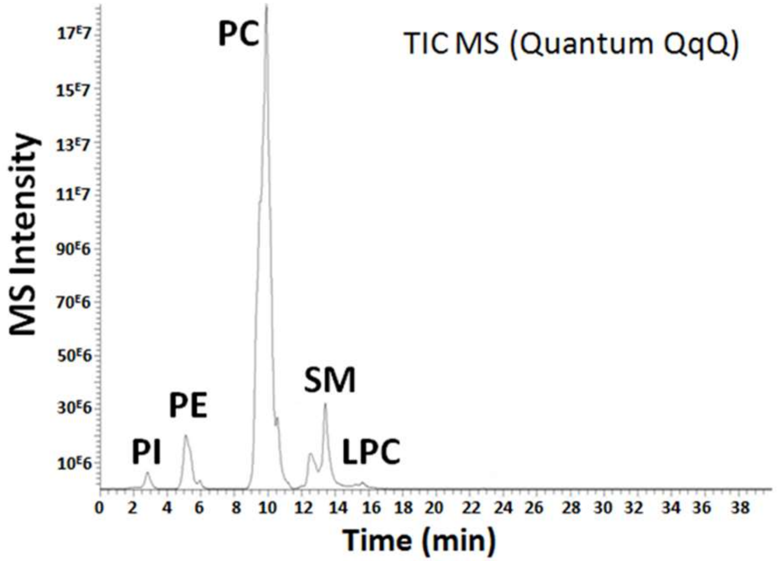

Analysis of phospholipids from a nasal mucosa sample using the triple quadrupole MS (Quantum) tuned to specific acquisition modes. PI species were analyzed by precursor ion scanning of m/z 241 in negative ionization. PE species were analyzed by neutral loss scanning of 141 Da in positive ion mode. PC, SM and LPC species were analyzed by precursor ion scanning of m/z 184 in positive ion mode. PS species were analyzed by neutral loss scanning of 185 Da in positive ionization. The analysis of PlsE was performed by MRM mode in negative ionization.

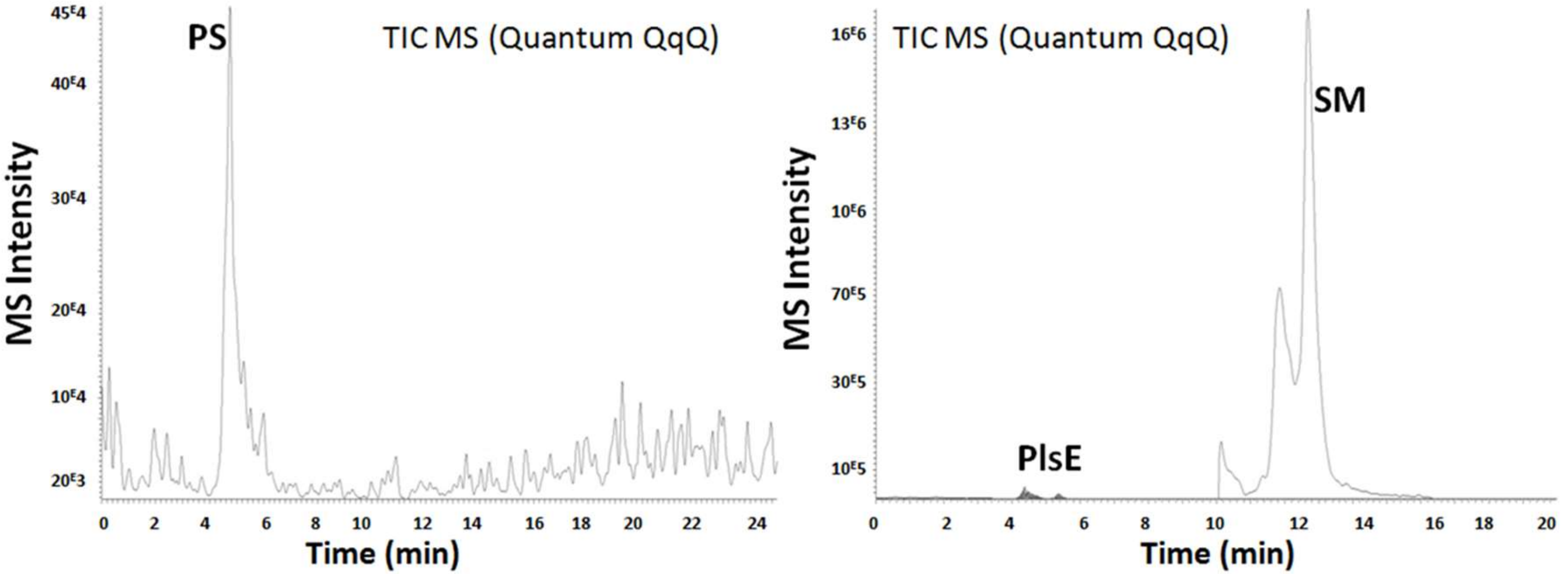

Supplementary Figure S3 (continued)

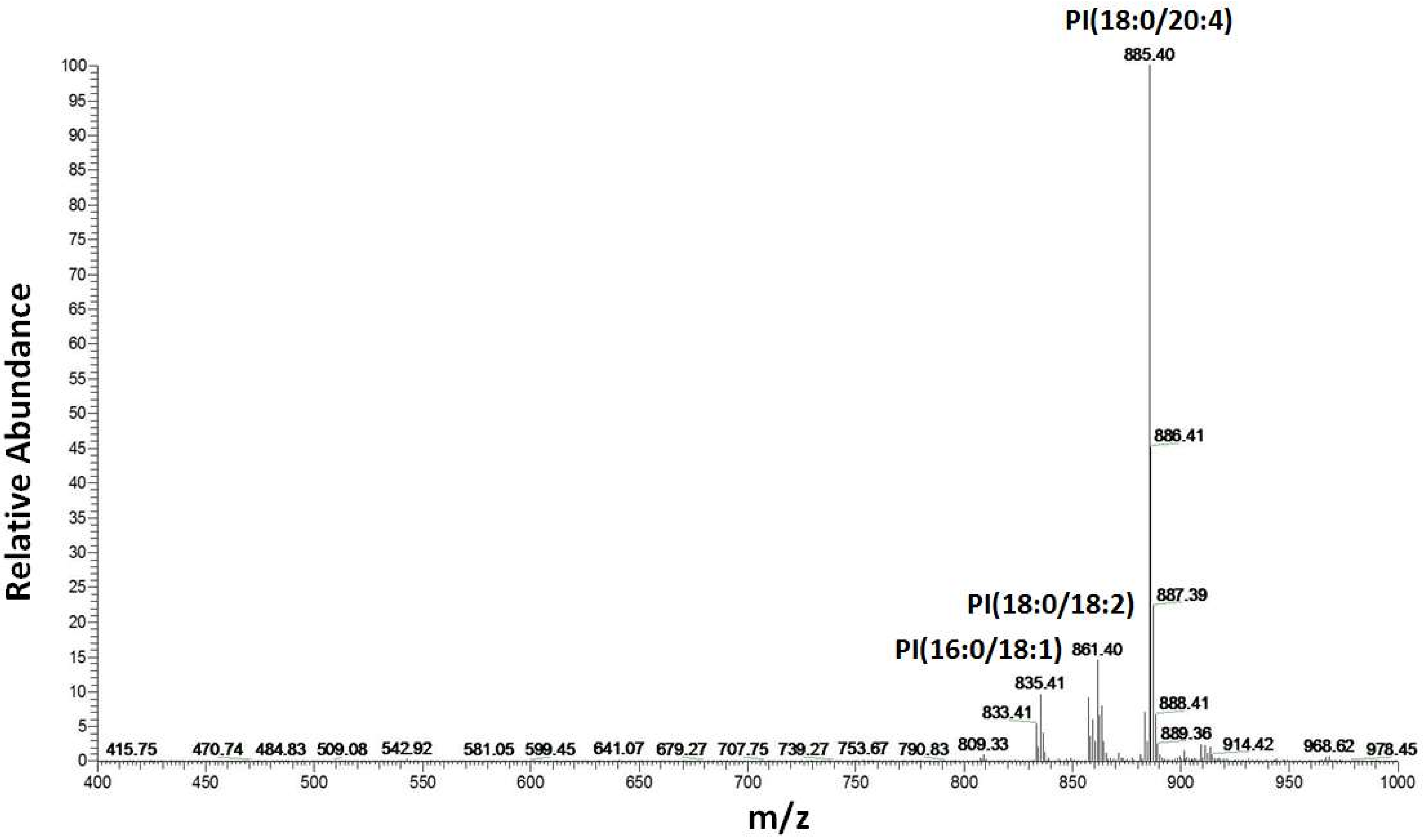

Mass spectrum of PI species from a nasal mucosa sample, after lipid extraction by the Folch method. Analysis of PI species was conducted by HPLC, under HILIC conditions, coupled to the triple quadrupole MS (Quantum) tuned to precursor ion scanning of m/z 241 in negative ionization.

Supplementary Figure S3 (continued)

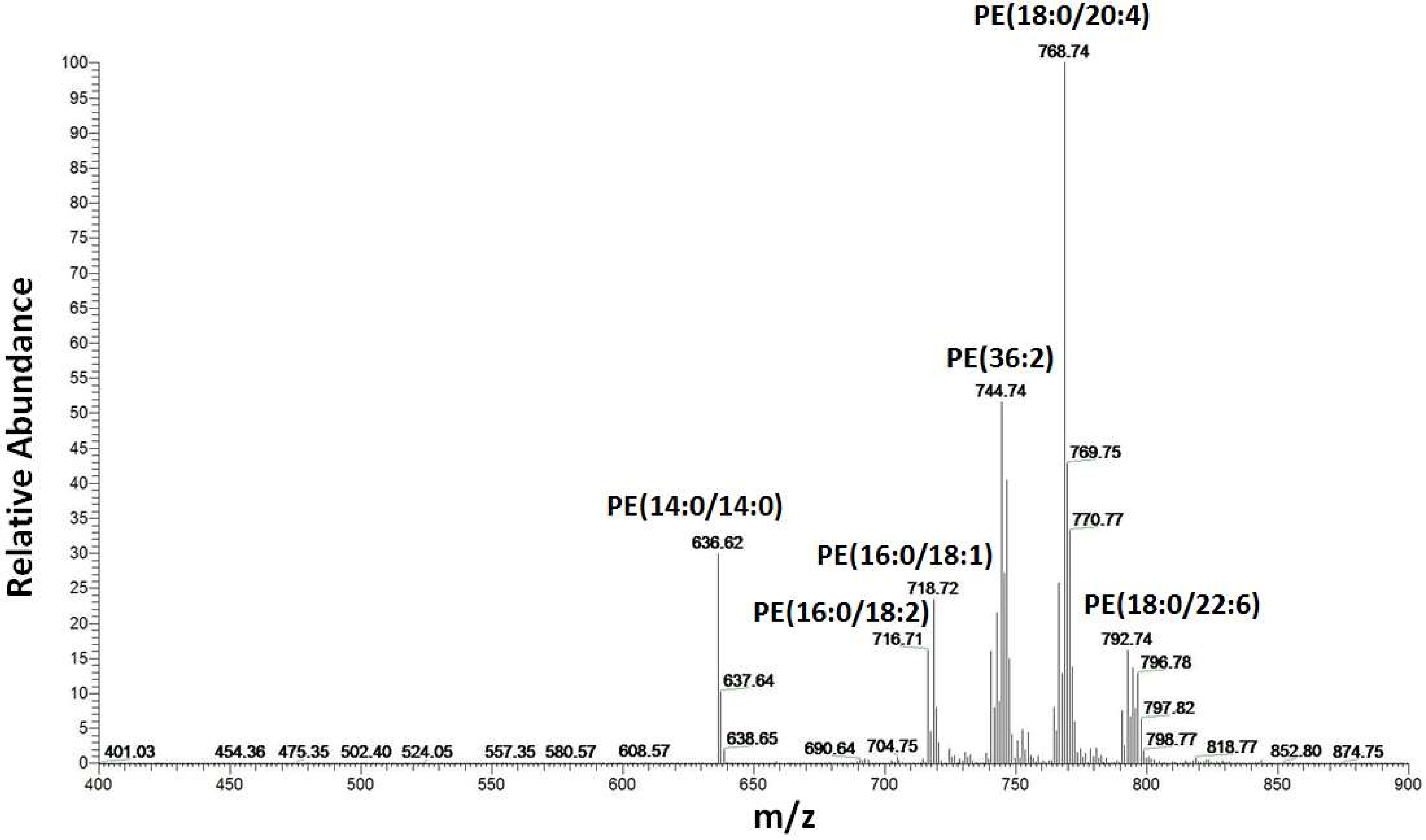

Mass spectrum of PE species from a nasal mucosa sample, after lipid extraction by the Folch method. Analysis of PE species was conducted by HPLC, under HILIC conditions, coupled to the triple quadrupole MS (Quantum) tuned to neutral loss scanning of 141 Da in positive ion mode.

Supplementary Figure S3 (continued)

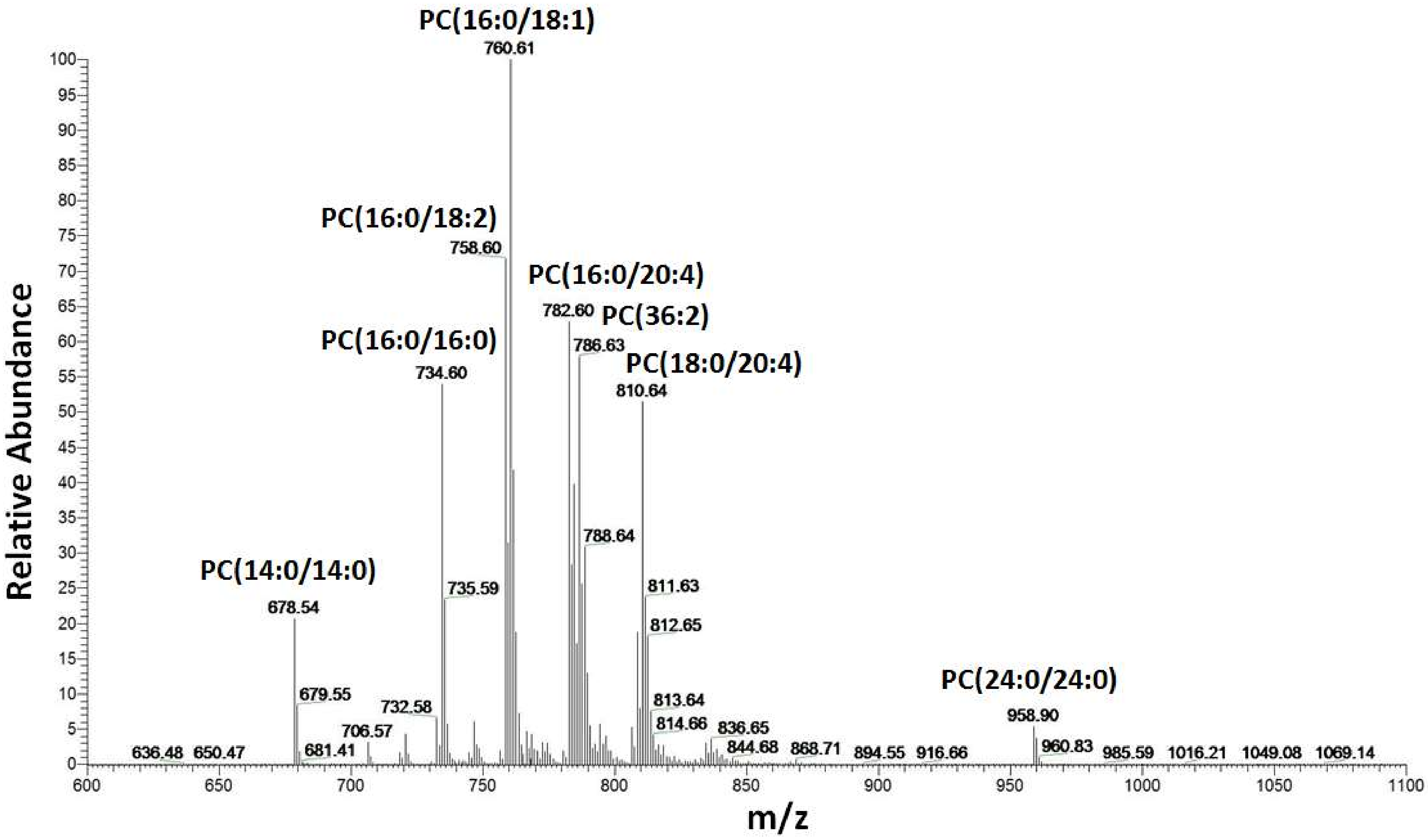

Mass spectrum of PC species in a nasal mucosa sample, after lipid extraction by the Folch method. Analysis of PC species was conducted by HPLC, under HILIC conditions, coupled to the triple quadrupole MS (Quantum) tuned to precursor ion scanning of m/z 184 in positive ion mode.

Supplementary Figure S3 (continued)

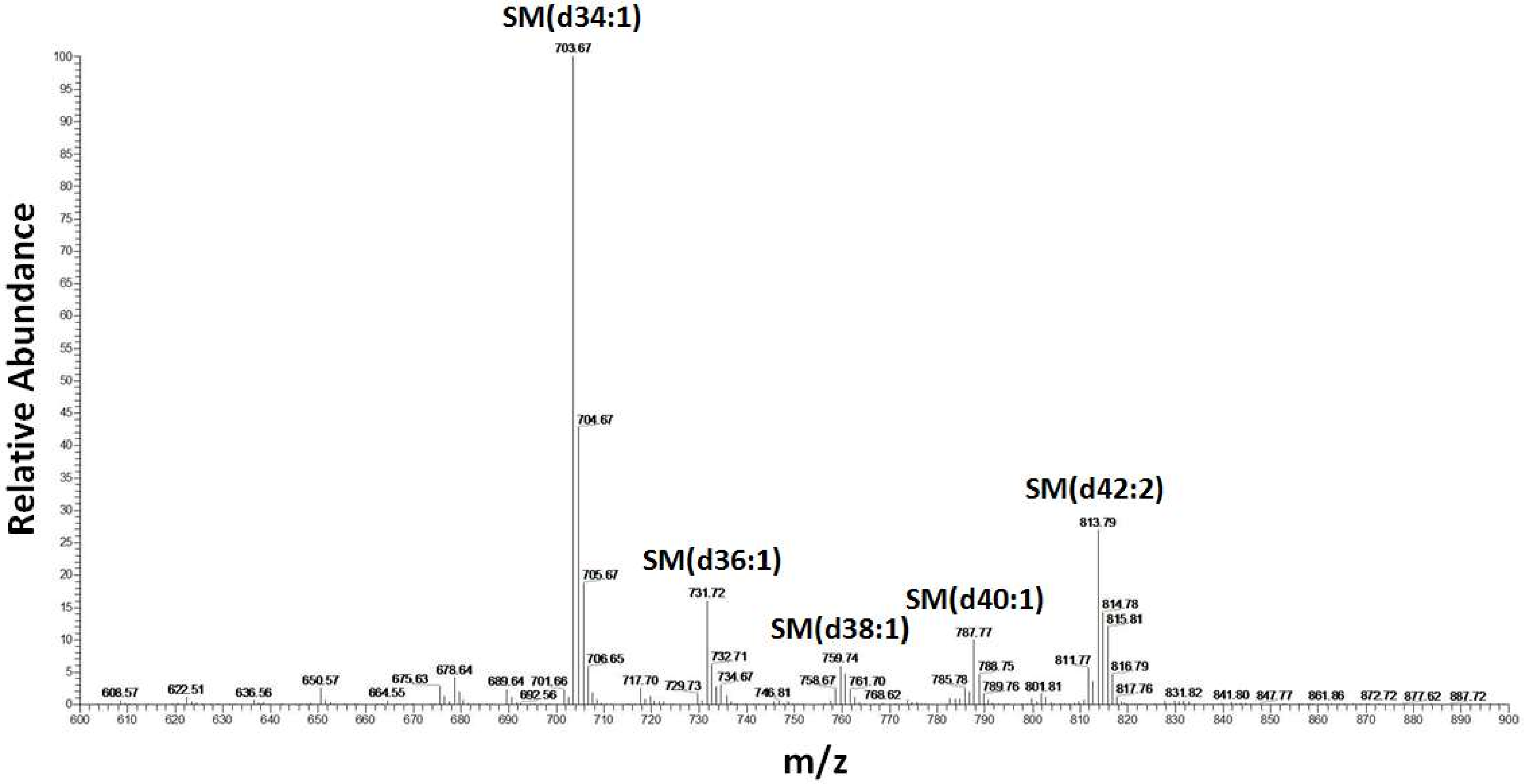

Mass spectrum of SM species from a nasal mucosa sample, after lipid extraction by the Folch method. Analysis of SM species was conducted by HPLC, under HILIC conditions, coupled to the triple quadrupole MS (Quantum) tuned to precursor ion scanning of m/z 184 in positive ion mode.

Supplementary Figure S3 (continued)

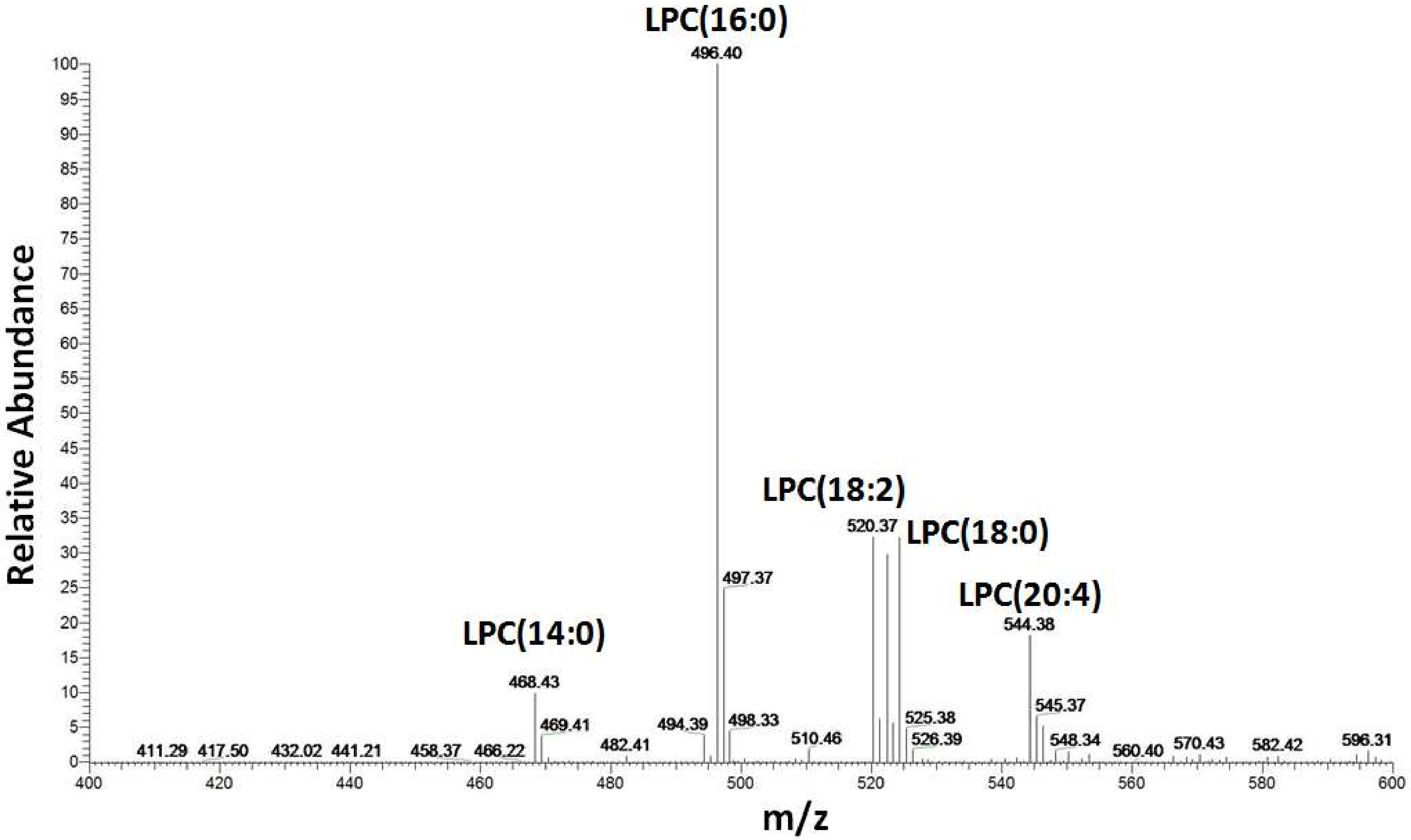

Mass spectrum of LPC species from a nasal mucosa sample, after lipid extraction by the Folch method. Analysis of LPC species was conducted by HPLC, under HILIC conditions, coupled to the triple quadrupole MS (Quantum) tuned to precursor ion scanning of m/z 184 in positive ion mode.

Supplementary Figure S3 (continued)

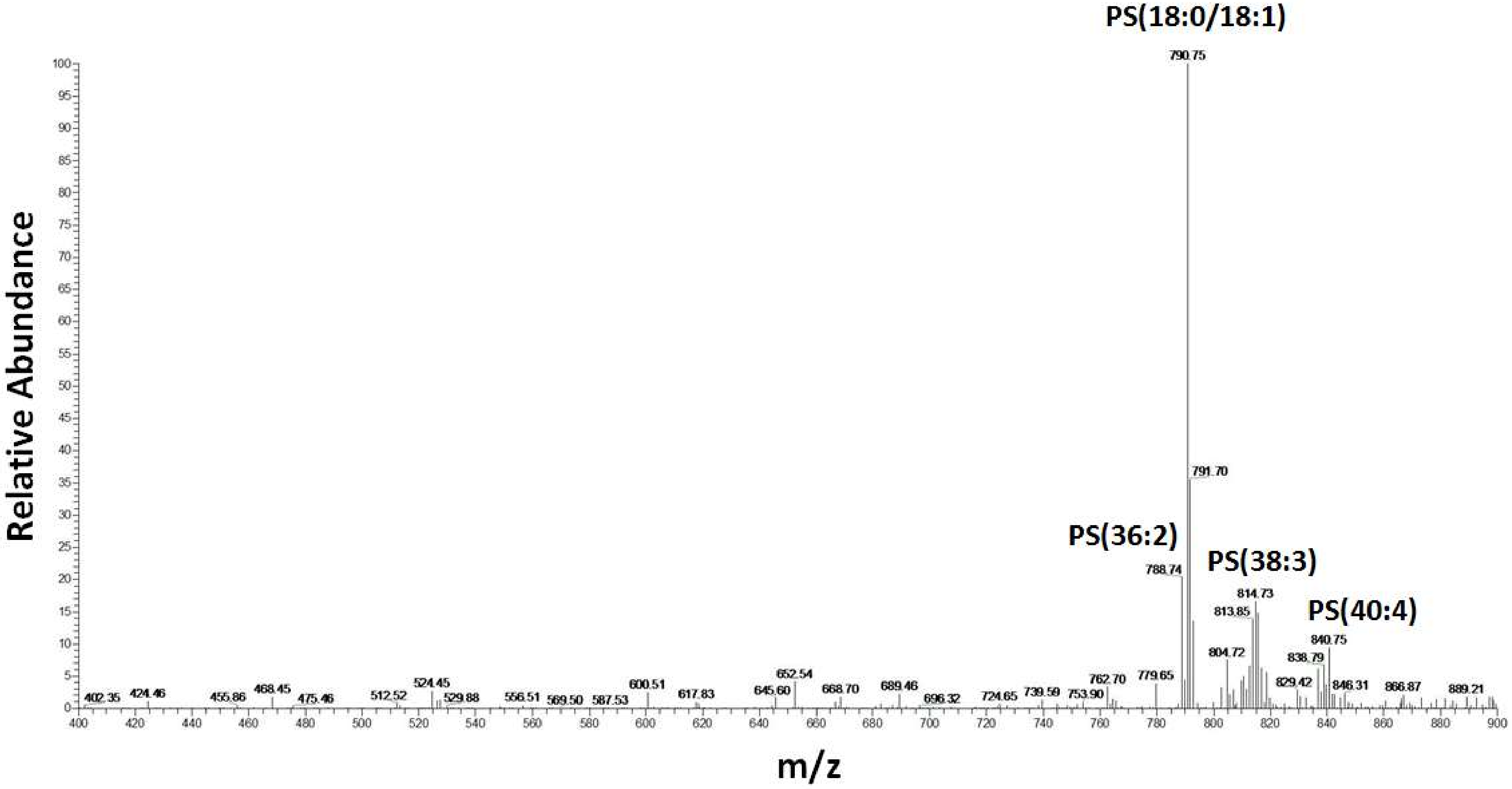

Mass spectrum of PS species from a nasal mucosa sample, after lipid extraction by the Folch method. Analysis of PS species was conducted by HPLC, under HILIC conditions, coupled to the triple quadrupole MS (Quantum) tuned to neutral loss scanning of 185 Da in positive ionization.
